# Syphilis Testing as a Proxy Marker for a Subgroup of Men Who Have Sex With Men With a Central Role in HIV-1 Transmission in Guangzhou, China

**DOI:** 10.3389/fmed.2021.662689

**Published:** 2021-07-07

**Authors:** Liping Huang, Hao Wu, Huanchang Yan, Yuanhao Liang, Qingmei Li, Jingwei Shui, Zhigang Han, Shixing Tang

**Affiliations:** ^1^Department of Epidemiology, School of Public Health, Southern Medical University, Guangzhou, China; ^2^Department of AIDS Control and Prevention, Guangzhou Center for Disease Control and Prevention, Guangzhou, China; ^3^Institute of Public Health, Guangzhou Medical University, Guangzhou, China; ^4^Wenzhou Institute, University of Chinese Academy of Sciences, Wenzhou, China

**Keywords:** syphilis, HIV-1, transmission network, screening, men who have sex with men

## Abstract

**Objectives:** The objectives of this study were to distinguish the role of men who have sex with men (MSM) with or without syphilis testing in HIV-1 transmission and to provide molecular evidence of syphilis testing as a proxy marker for identifying the subgroup of MSM.

**Methods:** HIV-1 transmission clusters were constructed by HIV-TRACE and Cluster Picker using HIV-1 pol sequences from 729 newly diagnosed HIV-infected MSM from 2008 to 2012 in Guangzhou, China. The role of MSM in HIV-1 transmission networks was determined by a node influence measurement and centrality analysis. The association between syphilis testing and factors related to HIV-1 transmission and antiretroviral treatment (ART) were analyzed by the Cox regression model.

**Results:** Among HIV-infected MSM, 56.7% did not test for syphilis at the time of HIV-1 diagnosis. MSM without syphilis testing was a specific subgroup of MSM with a larger closeness centrality and clustering coefficient than the recipients of syphilis testing (*P* < 0.001), indicating their central position in the HIV-1 transmission networks. The median degree and radiality within HIV-1 transmission networks as well as the median K-shell scores were also greater for MSM without syphilis testing (*P* < 0.001), suggesting their relatively greater contribution in transmitting HIV-1 than the receipts of syphilis testing. MSM with syphilis testing usually did not disclose their occupation or were more likely to be unemployed or to take non-skilled jobs, to have a history of sexually transmitted infections (STIs), and to be AIDS patients when diagnosed with HIV-1 infection (*P* < 0.05). Multivariable Cox regression analysis indicated that syphilis testing *per se* did not promote the engagement of ART (*P* = 0.233) or affect the speed of CD4^+^ T cell count recovery after treatment (*P* = 0.256).

**Conclusions:** Our study identifies syphilis testing as a proxy marker of a specific subgroup of HIV-infected MSM who refuse syphilis testing during HIV-1 diagnosis with an important role in HIV-1 transmission. Specific prevention and intervention targeting MSM without syphilis testing during HIV-1 care are urgently needed.

## Introduction

Over the past decades, syphilis has continued to spread globally, and disproportionately affects men who have sex with men (MSM) who are often co-infected with human immunodeficiency virus type one (HIV-1) (1–3). In light of the fact that syphilis and HIV-1 infection could facilitate the acquisition and transmission of each other, and even alter mutual disease progress (4–6), integrating syphilis testing into HIV-1 care has been recommended and promoted to curb the dual epidemic of syphilis and HIV-1 (7–9).

In China, despite the launch of a nationwide 10-year syphilis control plan in 2010 to promote the integration of HIV-1 and syphilis testing in the clinics of sexually transmitted infections (STIs) (10), there has been a continuous increase of syphilis cases and HIV-1/syphilis co-infections among MSM over the past decade (3, 11–13), due to low uptake of syphilis testing among HIV-infected MSM and other high-risk individuals in China (10, 13, 14). Furthermore, the current syphilis screening service might not fully cover all the risk groups. Hall et al. (13) have reported that MSM who did not engage in sex work were less likely to be tested for either HIV or syphilis. It is thus important to precisely determine the subgroup of MSM and to understand their difference in HIV-1 transmission (15). However, few studies have been published in China to investigate the role of MSM with or without syphilis testing in the control of HIV-1 epidemics (15), due to the lack of accurate and reliable information for MSM (16). Now, transmission network analysis using HIV-1 viral sequences has been revealed to be a valuable method to restore the social network of HIV-infected individuals in particular when epidemiological information, such as sexual behaviors, are not available or are inaccurate. The information obtained from HIV-1 transmission network topological structures may serve as a proxy for individuals with enhanced HIV-1 transmission risk (17, 18). For example, Ragonnet-Cronin et al. (19) adapted time-resolved phylogenetic analysis and HIV-1 transmission network analysis to identify 18.6% of all clustered heterosexual men as non-disclosed MSM in the UK. Based on HIV-1 transmission network analysis, we also found that non-disclosed MSM was a specific group and played a central role in HIV-1 transmission in China (20).

Therefore, in the current study, we adapted transmission network analysis to uncover the HIV-1 transmission risk among MSM with or without syphilis testing. We aimed to characterize the subgroup of MSM who refuse to test for syphilis and to provide molecular evidence of their important role in enhancing HIV-1 transmission in China.

## Materials and Methods

### Study Setting, Participants, and HIV-1 Sequences

Guangzhou is an international metropolis in the southern part of China, where the HIV-1 epidemic in MSM has been clustered for decades (21). The Center for Diseases Prevention and Control (CDC) is in charge of all HIV-1 cases in Guangzhou, including the diagnosis and confirmation of HIV-1 infection, CD4 cell measurement, and syphilis testing, etc. Eligibility criteria, recruitment, and measurement procedures for HIV-infected MSM have previously been described (22). Free consulting and testing for syphilis were available for all MSM at the time of HIV-1 testing or diagnosis, but they decided whether to take syphilis screening or not. Follow-up examinations were done every 3–6 months from June 2008 to February 2015. Demographic and laboratory testing data were collected from the medical charts of Guangzhou CDC. Blood samples were collected from 982 newly diagnosed and antiretroviral therapy (ART)-naïve HIV-1-infected MSM in Guangzhou CDC between January 2008 and December 2012. HIV-1 pol sequences (equal to the fragment of nucleotide 2,253–3,821 for the HIV-1 HXB2 strain) were successfully amplified by reverse transcription polymerase chain reaction (RT-PCR) and sequenced. The sequence data have been deposited in GenBank with the accession numbers listed in the Supplementary Material. Finally, a total of 729 (74.2%, 729/982) HIV-infected MSM were included in our study. The flow chart of the study is presented in Supplementary Figure 1. This study was approved by the Institutional Review Board of Guangzhou CDC (No. 2017030). Written informed consent was obtained from all the participants.

### Phylogenetic Analysis

HIV-1 pol sequences were aligned using the reference sequences of HIV-1 group M and the circulating recombinant forms (CRFs) obtained from the Los Alamos HIV-1 database (http://www.hiv.lanl.gov). HIV-1 genotypes were determined by the phylogenetic trees constructed by IQ-TREE 1.6.9 with 1000 ultrafast bootstrap replicates and 1000 replicates of the Shimodaira–Hasegawa approximate likelihood-ratio test (SH-aLRT) (23).

### Transmission Network Analysis

Transmission network analysis has been described previously (20). Briefly, all the HIV-1 sequences including a dataset of HIV-1 reference sequences from the Los Alamos National Laboratory HIV-1 database were aligned. The transmission clusters was identified by HIV Transmission Cluster Engine (HIV-TRACE) using a genetic distance threshold of 1.5% (24) or Cluster Picker with an intra-cluster genetic distance threshold of 4.5% (25, 26). The position of nodes in the transmission clusters were determined by nodal centrality indicators including degree, betweenness, closeness, clustering coefficient, and radiality, which were measured using NetworkAnalyzer 2.7 implemented in Cytoscape 3.7.0.13 (20, 27). K-shell (Ks) score, a measure of the cohesiveness of a subset of individuals among whom there are stronger, more direct, or more frequent ties than between other subgroups within the same network, was used to identify the hub node (28). Nodes with high Ks are more prone to infection and to be the most influential spreaders during epidemics (29–31). A novel graph theoretic clustering algorithm, Molecular Complex Detection (MCODE) implemented in Cytoscape 3.7.0.13, was adapted to identify the densely connected sub-networks in the large HIV-1 transmission clusters (32).

### Statistical Analysis

To compare the difference of the centrality metrics and Ks score of nodes between HIV-infected MSM with or without syphilis testing, the Wilcoxon signed-rank test was adapted to estimate the statistical significance in all the HIV-infected MSM or in 1:1 matched pairs of MSM generated by the propensity score matching (PSM) method (33, 34). We compared their epidemiological data at HIV-1 diagnosis and further adapted the logistic regression analysis to explore the association between syphilis testing and factors related to HIV-1 transmission. All the candidate variables with a *p*-value of <0.1 in the univariate model were sequentially included in a binary multivariate model using the stepwise forward method to estimate the association with syphilis testing. Multivariate Cox proportional hazards regression analysis was used to identify the independent factors associated with the engagement of ART and the speed of ART-related CD4^+^ T cell recovery. SPSS version 25.0 (SPSS Inc., Chicago, IL, USA) and R software version 4.0.2 were used to perform the statistical analysis.

## Results

### MSM Without Syphilis Testing Were More Often Located in the Big HIV-1 Transmission Clusters

A total of 729 HIV-1 sequences obtained from newly diagnosed HIV-infected MSM were used to construct HIV-1 molecular transmission networks using HIV-TRACE. Among them, 638 HIV-1 sequences were located in 76 clusters (Supplementary Figure 2A) and 27 (35.5%) clusters contained only 2 individuals. The proportion of cluster sizes of 3–5, 6–10, and >10 was 30.3, 15.8, and 18.4%, respectively (Supplementary Figure 2B). For the small clusters with fewer than 5 members, 70–82% of them contained at least one recipient of syphilis testing while only 44–48% of these clusters contained MSM without syphilis testing. In contrast, for big clusters with more than 6 members, the proportion of the clusters that contained MSM without syphilis testing dramatically increased and further reached similar levels to the clusters containing the recipients of syphilis testing (Supplementary Figure 2B). Furthermore, in the small clusters, the subjects were dominated by MSM with syphilis testing (62–63%) while the number of MSM with or without syphilis testing were almost equal in the big clusters (Supplementary Figure 2C). These results indicated that MSM without syphilis testing were more often located in the big clusters rather than in the small clusters of HIV-1 transmission.

Furthermore, Cluster Picker analysis identified 45 HIV-1 transmission clusters (Table 1, Supplementary Figure 3). MSM infected with HIV-1 CRF55_01B, CRF 01_AE, and CRF 07_BC were more likely to be clustered than subtype B or other HIV-1 genotypes (*p* < 0.05, Table 1). However, the composition of HIV-1 genotypes and the demographic characteristics of the clusters identified by HIV-TRACE and Cluster Picker were not different (Table 1).

**Table 1:** Demographic characteristics of HIV-infected MSM clustered by HIV-TRACE or Cluster Picker^&^.

**Characteristics**		**Total** **(*n* = 729, %)**	**HIV-TRACE (*****n*** **= 638)**		**Cluster picker (*****n*** **= 689)**	
			**Distribution of clustered sequences (%)**	**Percentage of clustered sequences (%)**	**Distribution of clustered sequences (%)**	**Percentage of clustered sequences (%)**
Screening for syphilis	Yes	316 (43.3)	284/638 (44.5)	284/316 (89.9)	300/689 (43.5)	300/316 (94.9)
	No	413 (56.7)	354/638 (55.5)	354/413 (85.7)	389/689 (56.5)	389/413 (94.2)
Age group (years)	16-30	429 (58.8)	384/638 (60.2)	384/429 (89.5)	411/689 (59.7)	411/429 (95.8)
	31-40	221 (30.3)	188/638 (29.5)	188/221 (85.1)	207/689 (30.0)	207/221 (93.7)
	≥41	79 (10.8)	66/638 (10.3)	66/79 (83.5)	71/689 (10.3)	71/79 (89.9)
Marital status	Single	517 (70.9)	452/638 (70.8)	452/517 (87.4)	489/689 (71.0)	489/517 (94.6)
	Married	165 (22.6)	149/638 (23.4)	149/165 (90.3)	156/689 (22.6)	156/165 (94.5)
	Divorced/separated/ widowed	47 (6.4)	37/638 (5.8)	37/47 (78.7)	44/689 (6.4)	44/47 (93.6)
Education level	Lower than senior high school	167 (22.9)	147/638 (23.0)	147/167 (88)	158/689 (22.9)	158/167 (94.6)
	Senior high school	232 (31.8)	208/638 (32.6)	208/232 (89.7)	219/689 (31.8)	219/232 (94.4)
	Junior college or higher	330 (45.3)	283/638 (44.4)	283/330 (85.8)	312/689 (45.3)	312/330 (94.5)
Occupation	Skilled/ professional work	428 (58.7)	371/638 (58.2)	371/428 (86.7)	402/689 (58.3)	402/428 (93.9)
	Unemployment/unskilled work	133 (18.2)	113/638 (17.7)	113/133 (85)	125/689 (18.1)	125/133 (94)
	Students	49 (6.7)	46/638 (7.2)	46/49 (93.9)	46/689 (6.7)	46/49 (93.9)
	Not disclosed	119 (16.3)	108/638 (16.9)	108/119 (90.8)	116/689 (16.8)	116/119 (97.5)
Ethnicity	Han	710 (97.4)	623/638 (97.6)	623/710 (87.7)	671/689 (97.4)	671/710 (94.5)
	Others	19 (2.6)	15/638 (2.4)	15/19 (78.9)	18/689 (2.6)	18/19 (94.7)
Number of sex partners in the past 6 months	≤ 1	210 (28.8)	186/638 (29.2)	186/210 (88.6)	201/689 (29.2)	201/210 (95.7)
	2 ~ 5	316 (43.3)	281/638 (44.0)	281/316 (88.9)	300/689 (43.5)	300/316 (94.9)
	>5	203 (27.8)	171/638 (26.8)	171/203 (84.2)	188/689 (27.3)	188/203 (92.6)
STIs history	Yes	101 (13.9)	83/638 (13.0)	83/101 (82.2)	96/689 (13.9)	96/101 (95)
	No	271 (37.2)	243/638 (38.1)	243/271 (89.7)	260/689 (37.7)	260/271 (95.9)
	Unknown	357 (49.0)	312/638 (48.9)	312/357 (87.4)	333/689 (48.3)	333/357 (93.3)
HIV-1 genotype	CRF 07_BC	265 (36.4)	263/638 (41.2)^*^	263/265 (99.2)^*^	263/689 (38.2)^*^	263/265 (99.2)^*^
	CRF 01_AE	263 (36.1)	224/638 (35.1)^*^	224/263 (85.2)^*^	260/689 (37.7)^*^	260/263 (98.9)^*^
	CRF 55_01B	115 (15.8)	112/638 (17.6)^*^	112/115 (97.4)^*^	110/689 (16.0)^*^	110/115 (95.7)^*^
	Subtype B	61 (8.4)	30/638 (4.7)	30/61 (49.2)	44/689 (6.4)^*^	44/61 (72.1)^*^
	Others	25 (3.4)	9/638 (1.4)^*^	9/25 (36.0)^*^	12/689 (1.7)	12/25 (48)
Baseline CD4^+^ counts (cell/mm^3^)	<200	111 (15.2)	92/638 (14.4)	92/111 (82.9)	104/689 (15.1)	104/111 (93.7)
	200 ~ 349	235 (32.3)	212/638 (33.3)	212/235 (90.2)	228/689 (33.1)	228/235 (97)
	350 ~ 499	229 (31.5)	200/638 (31.4)	200/229 (87.3)	216/689 (31.4)	216/229 (94.3)
	≥500	153 (21.0)	133/638 (20.9)	133/153 (86.9)	140/689 (20.3)	140/153 (91.5)
Disease stage^#^	HIV-1 infection	499 (68.4)	440/638 (69.0)	440/499 (88.2)	470/689 (68.2)	470/499 (94.2)
	AIDS	230 (31.6)	198/638 (31.0)	198/230 (86.1)	219/689 (31.8)	219/230 (95.2)

& *HIV-infected MSM were collected from 2008 to 2012 in Guangzhou, China*.

* *Statistically significant difference (P < 0.05, Chi-square tests) between clustered and non-clustered subjects*.

# *The definition and diagnosis of AIDS are based on the Guidelines for Diagnosis and Treatment of HIV/AIDS in China (2005)*.

*MSM, men who have sex with men; STI, sexually transmitted infection; HIV-1, human immunodeficiency virus type one; AIDS, acquired immune deficiency syndrome*.

### MSM Without Syphilis Testing Were More Efficient in HIV-1 Transmission

To further identify the key population driving HIV-1 transmission and to provide molecular evidence about the role of HIV-infected MSM without syphilis testing in HIV-1 transmission, we conducted centrality analysis and found that the following centrality indicators were significantly larger for MSM without syphilis testing than for the recipients of syphilis testing: the median degree (2.000, IQR 1.000–4.375 vs. 1.000, IQR 0.000–3.813, *P* = 0.004), the median radiality (1.000, 0.748–1.000 vs. 0.752, 0.000–1.000, *P* = 0.008), the clustering coefficient (0.645, 0.000–1.000 vs. 0.000, 0.000–0.792, *P* = 0.009), and the closeness centrality (1.000, 0.548–1.000 vs. 0.557, 0.000–1.000, *P* = 0.007), but no significant difference was observed for the betweenness centrality (*P* = 0.399, Table 2, Model 1**)**. To reduce the potential bias due to the heterogeneity between the MSM with or without syphilis testing, we further analyzed the centrality indicators using 1:1 matched pairs of the MSM with or without syphilis testing based on PSM methodology to adjust the potential differences of the baseline characteristics (Supplementary Table 1). Similar results were obtained (Table 2, Model 2**)**. These results indicated the central position of MSM without syphilis testing in the HIV-1 transmission networks and revealed their active role in HIV-1 transmission.

**Table 2:** Centrality analysis of HIV-1 transmission networks for MSM with or without syphilis testing during 2008-2012 in Guangzhou, China.

**Centrality indicators^a^**	**Syphilis screening (*****n*****, % or IQR, Model 1^b^)**			**Syphilis screening (*****n*****, % or IQR, Model 2^c^)**		
	**Yes (*n* = 413)**	**No (*n* = 316)**	***P***	**Yes (*n* = 220)**	**No (*n* = 220)**	***P*^d^**
Number clustered	284 (89.9)	354 (85.7)	0.092	188 (85.5)	186 (84.5)	0.789
Closeness centrality	0.557 (0.000, 1.000)	1.000 (0.548, 1.000)	0.007^**^	0.499 (0.000, 0.683)	0.742 (0.578, 0.917)	0.002^**^
Clustering coefficient	0.000 (0.000, 0.792)	0.645 (0.000, 1.000)	0.009^**^	0.556 (0.000, 0.768)	0.708 (0.564, 0.966)	0.017^**^
Degree	1.000 (0.000, 3.813)	2.000 (1.000, 4.375)	0.004^**^	2.500 (1.000, 5.667)	7.750 (4.000, 12.000)	0.004^**^
Radiality	0.752 (0.000, 1.000)	1.000 (0.748, 1.000)	0.008^**^	0.737 (0.000, 0.886)	0.905 (0.858, 0.944)	0.003^**^
Betweenness centrality	0.000 (0.000, 0.000)	0.000 (0.000, 0.003)	0.399	0.002 (0.000, 0.023)	0.006 (0.000, 0.109)	0.363
Ks of sub-networks	5.786 (0.000, 21.764)	7.000 (4.940, 21.700)	0.022^*^	2.392 (0.000, 6.693)	5.970 (4.000, 7.275)	0.011^*^
Number of seed nodes	1.0	7.0	0.069	1.0	2.0	–

a *The centrality indicators included closeness (the speed to spread from a given node to other reachable nodes or the sum of the distances from a given node to all other reachable nodes in the network), clustering coefficient (the density of the immediate neighborhood or the level of interconnection between members of a node's neighboring nodes), degree (the number of edges linked to a node or the complex of connectivity), radiality (accessibility to other nodes), betweenness (the extent of influence for a subject in facilitating communication between pairs of subjects, which is defined as the fraction of shortest paths going through a given node), Ks (K-shell score, a measure of the cohesiveness of a subset of individuals among whom there are stronger, more direct, or more frequent ties than between other subgroups within the same network) and seeds (the highest scoring node in the cluster). Data are the median and their interquartile range (IQR) for the abnormal distribution of indicators*.

b *Model 1: Comparison and analysis were conducted in the two groups of MSM with or without syphilis screening among all the HIV-1 transmission clusters*.

c *Model 2: Comparison and analysis were conducted between the clusters with propensity score-matched pairs of MSM with or without syphilis screening*.

d *Statistical significance was calculated using a Wilcoxon signed-rank test. A P < 0.05 is statistically significant*.

* *P < 0.05;*

** *P < 0.01*.

**Figure 1:**
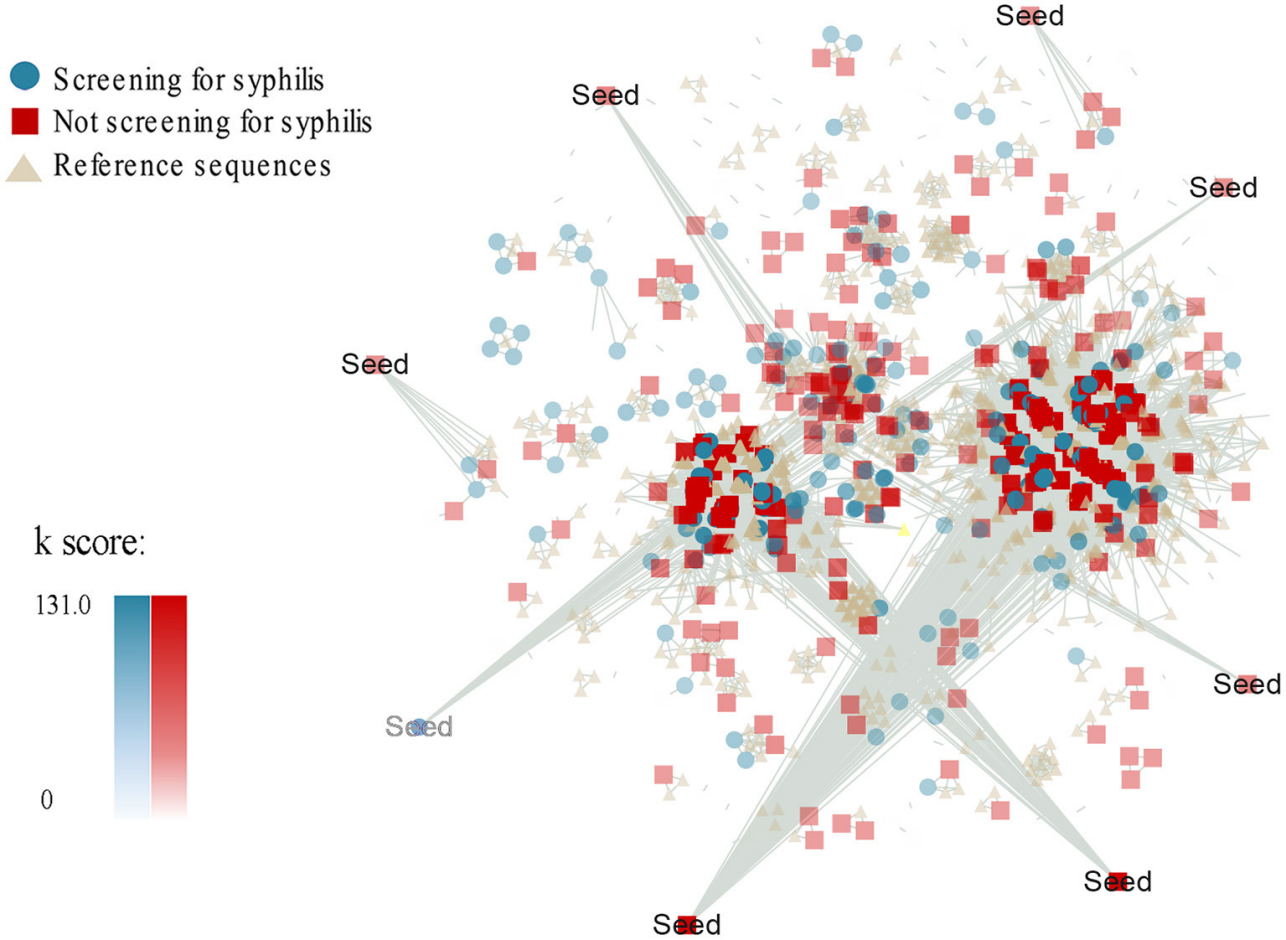
Ks score-associated and group-associated genetic transmission networks for MSM with or without syphilis testing during 2008-2012 in Guangzhou, China. Ks score was measured by a K-shell decomposition algorithm. The colors and shapes represent different groups of the HIV-1 sequences from the MSM with syphilis screening (green circle) or without syphilis screening (red square) and the HIV-1 reference sequences (light yellow triangle). The density of the colors indicates the Ks score values. Seed is the highest scoring node in the densely connected sub-networks.

Furthermore, we adapted a node influence measurement by calculating the Ks score of a node to identify the most influential spreaders in the HIV-1 transmission networks. Interestingly, we found that median Ks scores were statistically greater for the MSM without syphilis testing than those with syphilis testing (7.000, IQR 4.940–21.700 vs. 5.786, IQR 0.000–21.764, *P* = 0.022, Table 2, Figure 1). Then, we used MCODE analysis to analyze the densely connected sub-networks in the large transmission clusters and identified 33 key sub-networks and 8 seed nodes (Figure 1). Of note, the seed nodes were more frequently identified to be MSM without syphilis testing than those with syphilis screening (7/8 vs. 1/8) although the difference did not reach statistical significance (*P* = 0.069, Table 2, Model 1**)**. These results reiterated that MSM without syphilis testing may be a specific subgroup of MSM with more efficient HIV-1 transmission.

### MSM With and Without Syphilis Testing Were Different Subgroups

Although free syphilis testing was provided upon the diagnosis of HIV-1 infection, in our study, 56.7% of HIV-infected MSM refused syphilis testing at the time of HIV-1 diagnosis. For those with or without syphilis testing, the significant differences were observed with regard to the occupation, the number of sex partners in the past 6 months, the history of STIs, and the stage of HIV-1 infection (Table 3). For example, 20.1% of the MSM without syphilis testing were unemployed or took non-skilled jobs, and 23.7% of them did not report their occupation while 77.5% of the recipients of syphilis testing had skilled/professional jobs or were students (*P* < 0.001). Furthermore, 42.4% of MSM without syphilis testing reported only one sex partner during the past 6 months whereas multiple sex partners were recorded in 88.9% of MSM with syphilis testing (*p* < 0.001). In addition, acquired immunodeficiency syndrome (AIDS) was recorded slightly more frequently in the MSM without syphilis testing than the recipients of syphilis testing (35.6 vs. 26.6%, *p* = 0.012). The percentage of past STIs was also different between MSM with or without syphilis testing (*p* < 0.001) in part due to the difference in regard to the awareness of their STIs (57.6 vs. 42.4%, *p* = 0.001, Supplementary Table 2). Multivariable logistic regression analysis also confirmed that MSM who did not report their occupations were most unlikely to receive syphilis testing [adjusted odds ratio (aOR) = 4.468, *p* < 0.001, Table 4]. In addition, syphilis testing was less likely to be accepted by those who disclosed their history of STIs than those who did not know their STIs (aOR = 0.466, *p* = 0.001, Table 4), Furthermore, MSM without syphilis testing were less likely to have multiple sex partners (*p* < 0.001, Table 4). Of note, the distribution of ethnicity (*p* = 0.129) and HIV-1 genotypes (*p* = 0.316) were not statistically different between MSM with and without syphilis testing (Table 3).

**Table 3:** Comparison of demographic characteristics for HIV-infected MSM with or without syphilis screening from 2008 to 2012 in Guangzhou, China.

**Characteristics**		**Syphilis screening (*****n*****, %)**		***P*-value**
		**Yes (*n* = 316)**	**No (*n* = 413)**	
Age group (years)	16-30	193 (61.1)	236 (57.1)	0.528^d^
	31-40	92 (29.1)	129 (31.2)	
	≥41	31 (9.8)	48 (11.6)	
Marital status	Single	219 (69.3)	298 (72.2)	0.693^e^
	Married	76 (24.1)	89 (21.5)	
	Divorced/separated/widowed	21 (6.6)	26 (6.3)	
Education level	Lower than senior high school	78 (24.7)	89 (21.5)	0.434^e^
	Senior high school	103 (32.6)	129 (31.2)	
	Junior college or higher	135 (42.7)	195 (47.2)	
Occupation	Skilled/professional work	219 (69.3)	209 (50.6)	<0.001^***^^e^
	Unemployment/unskilled work	50 (15.8)	83 (20.1)	
	Students	26 (8.2)	23 (5.6)	
	Not disclosed	21 (6.6)	98 (23.7)	
Ethnicity	Han	331 (98.4)	339 (96.6)	0.129
	Others	5 (1.6)	14 (3.4)	
Number of sex partners in the past 6 months	≤ 1	35 (11.1)	175 (42.4)	<0.001^***^^d^
	2~5	148 (46.8)	168 (40.7)	
	>5	133 (42.1)	70 (16.9)	
STIs history	Yes	35 (11.1)	66 (16.0)	<0.001^***^^e^
	No	99 (31.3)	172 (41.6)	
	Unknown	182 (57.6)	175 (42.4)	
HIV-1 genotype	CRF 07_BC	128 (40.5)	137 (33.2)	0.316^e^
	CRF 01_AE	109 (34.5)	154 (37.3)	
	CRF 55_01B	47 (14.9)	68 (16.5)	
	Subtype B	22 (7.0)	39 (9.4)	
	Others	10 (3.2)	15 (3.6)	
Baseline CD4^+^ counts (cell/mm^3^)	<200	43 (13.6)	69 (16.7)	0.184^d^
	200~349	95 (30.1)	140 (33.9)	
	350~499	101 (32.0)	128 (31.0)	
	≥500	77 (24.4)	76 (18.4)	
Disease stage^a^	HIV-1 infection	232 (73.4)	267 (64.6)	0.012^*^^e^
	AIDS	84 (26.6)	146 (35.4)	
ART initiation after diagnosis^b^	Yes	156 (55.5)	249 (69.4)	<0.001^***^^e^
	No	125 (44.5)	110 (30.6)	
Median time from HIV-1 diagnosis to ART initiation (IQR, years)		3.0 (1.2–3.7)	2.5 (0.7–3.7)	0.037^*^^f^
CD4^+^ T cell count recovery after ART^c^	Yes	78 (84.8)	103 (73.6)	0.044^*^^e^
	No	14 (15.2)	37 (26.4)	
Median time to CD4^+^ T cell count recovery (IQR, months)^c^		5.1 (1.8–8.5)	9.1 (4.3–13.9)	0.124^f^

a *The definition and diagnosis of AIDS are based on the Guidelines for Diagnosis and Treatment of HIV/AIDS in China (2005)*.

b *ART-naïve MSM with at least one follow-up visit data (n = 640)*.

c *CD4^+^ T cell recovery is defined as the increase of CD4^+^ cell count from <350 before ART to >350 cells/mm after ART*.

d *Kruskal-Wallis tests;*

e *Chi-square tests;*

f *Mann-Whitney U-tests. A P < 0.05 is statistically significant.*

* *P < 0.05;*

*** *P < 0.001*.

*MSM, men who have sex with men; STI, sexually transmitted infection; HIV-1, human immunodeficiency virus type one; AIDS, acquired immune deficiency syndrome*.

**Table 4:** Factors associated with no syphilis testing among HIV-infected MSM from 2008 to 2012 in Guangzhou, China.

**Characteristics**		**Unadjusted *P*-value^a^**	**Unadjusted OR**	**90% C.I**.	**Adjusted *P*-value^b^**	**Adjusted OR**	**95% C.I**.
Occupation	Skilled/professional work	<0.001	Reference		<0.001	Reference	
	Unemployment/unskilled work	0.007	1.739	1.245, 2.431	0.080	1.483	0.954, 2.306
	Students	0.802	0.927	0.564, 1.524	0.446	0.778	0.408, 1.484
	Not disclose	<0.001	4.890	3.193, 7.489	<0.001	4.468	2.602, 7.675
Number of sexual partners in the past 6 months	≤ 1	<0.001	Reference		<0.001	Reference	
	2~5	0.004	1.689	1.254, 2.274	<0.001	0.192	0.123, 0.302
	>5	<0.001	3.799	2.308, 6.254	<0.001	0.097	0.060, 0.159
STIs history	Known	<0.001	Reference		<0.001	Reference	
	Unknown	<0.001	0.541	0.422, 0.694	<0.001	0.466	0.332, 0.655
Disease stage	HIV-1 infection	0.012	Reference		0.072	Reference	
	AIDS	0.012	1.510	1.154, 1.977	0.072	1.392	0.970, 1.997

a *The univariate logistic regression model. A P < 0.1 is statistically significant*.

b *The multivariate logistic regression model. A P < 0.05 is statistically significant*.

*MSM, men who have sex with men; STI, sexually transmitted infection; HIV-1, human immunodeficiency virus type one; AIDS, acquired immune deficiency syndrome; OR, odds ratio; C.I., confidence interval*.

### Syphilis Testing Was Not Associated With ART Initiation and ART-Related CD4 Cell Recovery

**Figure 2:**
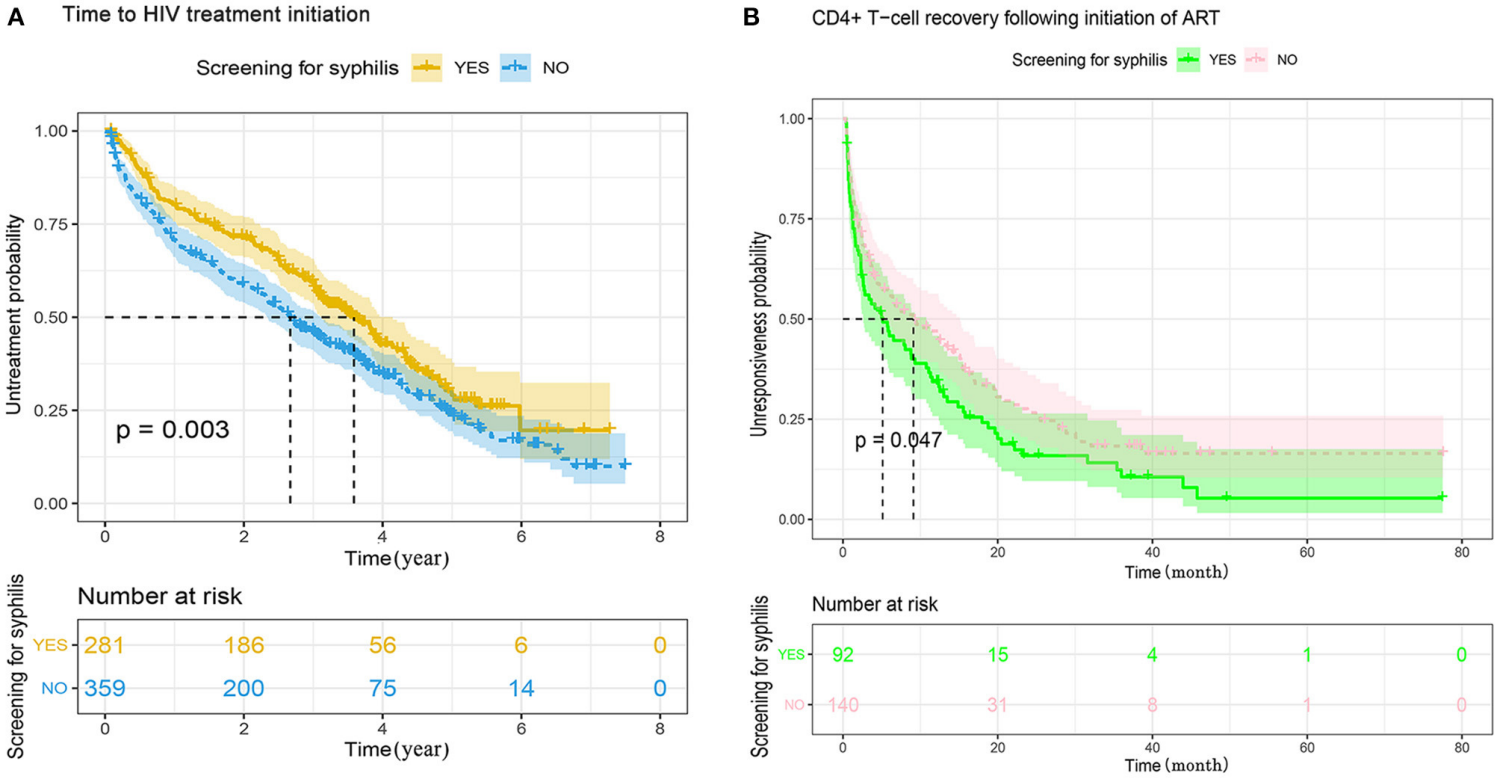
**(A)** Kaplan–Meier analysis of the time to initiate antiretroviral treatment between MSM with or without syphilis screening in Guangzhou, China, 2008–2012. The date of HIV-1 diagnosis was defined as time zero. The tick marks indicate the participants with censored data. *P*-values were calculated by the log-rank test. The median time to initiate ART was 2.7 years (range 2.3-3.0) for MSM without syphilis testing, and 3.6 years (range 3.1-4.1) for MSM with syphilis screening, respectively. MSM, men who have sex with men; HIV, human immunodeficiency virus. **(B)** Kaplan–Meier analysis of the time from CD4^+^ count <350–350 cells/μL following ART between MSM with or without syphilis screening. The date of ART initiation was defined as time zero. The tick marks indicate the participants with censored data. *P*-values were calculated by the log-rank test. The median time from CD4^+^ T-cell count <350–≥350 cells/μL was 9.1 months (range 4.3-13.9) for the patients without syphilis testing, and 5.1 months (range 1.8-8.5) for the patients with syphilis screening.

To investigate the difference between MSM with or without syphilis testing in the compliance of HIV-1 care, in particular the engagement of ART and the response to ART, we summarized the baseline characteristics between the subjects with or without ART (Supplementary Table 3), and found that the percentage of ART engagement was slightly higher in those without syphilis testing than the recipients of syphilis testing (69.4 vs. 55.5%, *P* = 0.001, Supplementary Table 3). Kaplan–Meier analysis also indicted that MSM without syphilis testing started ART earlier than the recipients of syphilis testing (*P* = 0.003, Figure 2A). To further explore the reasons for the difference of ART initiation, we adapted multivariate Cox proportional hazards regression analysis and found that syphilis testing was not an independent factor associated with ART initiation (adjusted *p* = 0.233, Table 5). The factors associated with the time to initiate ART included age, HIV-1 genotypes, the time of HIV-1 diagnosis, baseline CD4^+^ cell count, disease stage during HIV-1 diagnosis, and the disclosure of STIs (Table 5). Because STI history was associated with both syphilis testing with an unadjusted OR of 0.541 (Table 4) and ART initiation (unadjusted HR = 0.713, Table 5), we stratified the MSM population according to the disclosure of STIs in a time-to-event model to avoid confounding the STIs. The result indicated that ART engagement was not affected by syphilis testing in those aware (*P* = 0.546) or unaware of their STI history (*P* = 0.141, Supplementary Figure 4). These results indicated that syphilis testing *per se* did not affect the initiation of ART among HIV-infected MSM.

**Table 5:** Factors associated with the time from HIV-1 diagnosis to antiretroviral treatment initiation between HIV-infected MSM with or without syphilis screening during 2008 and 2012 in Guangzhou, China.

**Characteristics**		**Unadjusted *P*^a^**	**Unadjusted HR**	**90% C.I**.	**Adjust *P*^b^**	**Adjust HR**	**95% C.I**.
Screening for syphilis	Yes	0.003	Reference		0.233	Reference	
	No	0.003	1.353	1.143, 1.600	0.233	1.139	0.919, 1.412
Age group (years)	16-30	0.002	Reference		0.032	Reference	
	31-40	0.026	1.283	1.068, 1.543	0.011	1.381	1.076, 1.773
	≥41	0.001	1.62	1.264, 2.075	0.079	1.398	0.962, 2.032
Marital status	Single	0.005	Reference		0.655	Reference	
	Married	0.001	1.446	1.197, 1.747	0.734	1.051	0.789, 1.401
	Divorced/separated/widowed	0.362	1.206	0.860, 1.690	0.358	1.230	0.791, 1.910
Occupation	Skilled/professional work	0.025	Reference		0.278	Reference	
	Unemployment/unskilled work	0.014	1.375	1.112, 1.700	0.167	1.202	0.926, 1.559
	Students	0.873	0.966	0.679, 1.376	0.617	1.118	0.722, 1.731
	Not disclose	0.026	1.349	1.082, 1.682	0.087	1.275	0.966, 1.683
STIs history	Known	0.001	Reference		0.030	Reference	
	Unknown	0.001	0.713	0.604, 0.842	0.030	0.795	0.646, 0.978
HIV-1 genotypes	CRF 07_BC	<0.001	1		0.008	Reference	
	CRF 01_AE	0.001	1.475	1.216, 1.789	0.056	1.269	0.994, 1.619
	CRF 55_01B	0.566	0.914	0.708, 1.182	0.119	0.779	0.570, 1.066
	Subtype B	0.002	1.777	1.304, 2.420	0.089	1.389	0.951, 2.030
	Others	0.328	1.305	0.834, 2.044	0.182	1.445	0.841, 2.483
Baseline CD4^+^ counts (cell/mm^3^)	<200	<0.001	Reference		<0.001	Reference	
	200–350	0.079	0.782	0.621, 0.985	0.222	1.214	0.889, 1.658
	351–500	<0.001	0.456	0.359, 0.578	0.355	0.844	0.589, 1.209
	≥500	<0.001	0.226	0.167, 0.304	<0.001	0.417	0.272, 0.639
Disease stage	HIV-1 infection	<0.001	Reference		<0.001	Reference	
	AIDS	<0.001	0.391	0.331, 0.461	<0.001	1.847	1.415, 2.410

a *The univariate Cox proportional hazards regression model. A P < 0.1 is statistically significant*.

b *The multivariate Cox proportional hazards regression model. A P < 0.05 is statistically significant*.

*MSM, men who have sex with men; STI, sexually transmitted infection; HIV, human immunodeficiency virus; AIDS, acquired immune deficiency syndrome; HR, hazard ratio; C.I., confidence interval*.

Furthermore, the speed of CD4^+^ T cell recovery following ART was analyzed among the subjects with baseline CD4^+^ T cell count <350 cell/mm^3^ and at least two follow-up visit records. Among the 232 HIV-infected MSM analyzed, CD4^+^ cell count increased to ≥350 cells/mm^3^ in 84.8% (78/181) and 73.6% (103/181) of the subjects with or without syphilis testing, respectively. However, the difference was marginally significant (*P* = 0.044, Supplementary Table 4). Kaplan-Meier analysis indicated that HIV-infected MSM with syphilis testing achieved CD4^+^ cell count >350 cells/mm^3^ significantly earlier than the patients without syphilis testing. The median time was 5.1 months for the recipients of syphilis testing and 9.1 months for those without syphilis testing, respectively (*P* = 0.047, Figure 2B). Multivariate Cox proportional hazards regression analysis indicated that syphilis testing did not directly affect the outcome of CD4^+^ T cell recovery (*P* = 0.256, Table 6). In contrast, the speed of CD4^+^ T cell recovery was mainly associated with baseline CD4^+^ T cell counts at the time of HIV-1 diagnosis (*P* < 0.001) or ART initiation (*P* < 0.001, Table 6).

**Table 6:** Factors associated with the time to CD4^+^ T-Cell recovery following initiation of ART between HIV-infected MSM with or without syphilis screening during 2008 and 2012 in Guangzhou, China.

**Characteristics**		**Unadjusted *P*^a^**	**Unadjusted HR**	**90% C.I**.	**Adjust *P*^b^**	**Adjust HR**	**95% C.I**.
Screening for syphilis	Yes	0.048	Reference		0.256	Reference	
	No	0.048	0.743	0.580, 0.951		0.841	0.623, 1.134
HIV-1 genotype	CRF 07_BC	0.006	Reference		0.078	Reference	
	CRF 01_AE	0.001	0.596	0.457, 0.776	0.027	0.696	0.506, 0.959
	Subtype B	0.192	0.740	0.506, 1.082	0.691	0.911	0.577, 1.439
Disease stage	HIV-1 infection	<0.001	Reference		0.476	Reference	
	AIDS	<0.001	0.271	0.209, 0.352		0.870	0.593, 1.276
CD4^+^ counts at HIV diagnosis (cell/mm^3^)	<200	<0.001	Reference		<0.001	Reference	
	200–350	<0.001	3.573	2.478, 5.150	0.013	1.922	1.149, 3.214
	351–500	<0.001	8.447	6.229, 14.326	<0.001	3.841	2.15, 6.862
CD4^+^ counts at ART initiation (cell/mm^3^)	<200	<0.001	Reference		<0.001	Reference	
	200–350	<0.001	4.918	3.651, 6.626	<0.001	2.874	1.851, 4.464
Treatment regimen^c^	NVP+3TC+AZT	0.894	Reference				
	EFV+3TC+AZT	0.858	1.042	0.716, 1.517			
	EFV+3TC+TDF	0.668	0.911	0.638, 1.302			
	Others	0.654	0.910	0.643, 1.287			

a *The univariate Cox proportional hazards regression model. A P < 0.1 is statistically significant*.

b *The multivariate Cox proportional hazards regression model. A P < 0.05 is statistically significant*.

c *According to the manual for China's National Free Antiretroviral Therapy. 2nd ed. Beijing, China: People's Medical Publishing House 2008*.

*MSM, men who have sex with men; HIV, human immunodeficiency virus; AIDS, acquired immune deficiency syndrome; ART, antiretroviral therapy; 3TC, Lamivudine; AZT, Zidovudine; TDF, Tenofovir; EFV, Efavirenz; HR, hazard ratio; C.I., confidence interval*.

## Discussion

Syphilis screening is important for the control of both syphilis and HIV-1 infection. However, a large number of HIV-positive MSM do not receive syphilis testing as recommended (10, 14, 15). In our study, 56.7% of newly diagnosed HIV-infected MSM refused to take syphilis testing although it is provided for free in China. Our results are in line with the data obtained from HIV-infected MSM in Shanghai, China where the rate of syphilis testing was approximately 50% in 2010 (13). A recent online national survey in China also revealed that the acceptance rate of syphilis self-testing was 51.7% among MSM (35). Of note, the syphilis testing rate was suboptimal in the United States where only 68.3% of HIV-positive MSM took this testing during 2017–2018 (36). Though several studies have presented the socio-demographic characteristics of MSM with and without syphilis testing uptake (13, 35, 37), few studies investigated the impact of MSM who refuse to take syphilis testing on HIV-1 transmission and ART treatment partly because of the difficulty of identifying the subgroups of HIV-infected persons and precisely tracing their HIV-1 transmission.

Fortunately, HIV-1 genetic network analysis has been well-documented to be able to identify the subgroup of persons with specific significance in HIV-1 transmission, and to provide precise prevention measures targeting the specific sub-population (38, 39). In our study, we found that the MSM without syphilis testing were more likely to be found in the center of HIV-1 transmission networks and had a more intimate and direct relationship with other MSM according to their greater degree, radiality, and clustering coefficient within HIV-1 transmission networks. These results indicated that they were prone to contribute to the small world properties rather than in an intermediary form (40–42). Well-documented small world effect is characteristic of fast and efficient information transfer (40, 43). The presence of small world effect might be a mirror of efficient HIV-1 transmission among the HIV-infected MSM population who refuse to receive syphilis testing. Furthermore, coreness centrality obtained in the K-shell decomposition process is well-known to be a better measure than the degree to identify the potential influential spreaders in the transmission network (29–31). In our study, K-shell decomposition analysis also confirmed MSM who refuse syphilis testing as a specific subgroup of HIV-infected subjects with an active role in HIV-1 transmission. To our best knowledge, this is the first study to adapt HIV-1 transmission network analysis to characterize MSM without syphilis testing and to provide molecular evidence of their important role in enhancing HIV-1 transmission in China.

Our results indicated that such a specific group of MSM may lack knowledge about HIV-1 and syphilis or may not want to test for syphilis due to personal reasons (13). For example, in our study, MSM without syphilis testing were more likely to be unemployed or take non-skilled jobs, previous studies also indicated that non-testing of syphilis was more common among MSM with low HIV knowledge and lower income (13). Of note, we found that 49% of HIV-infected MSM did not know if they had STIs at the time of HIV-1 diagnosis. Several studies have shown that most persons with syphilis, including up to 44% of MSM, are unaware of their serostatus of syphilis as they may be asymptomatic for years (44, 45) or unwilling to disclose their STI diagnosis (46) and their sexual behaviors (15, 47). Our results also showed that MSM without syphilis screening were more likely to be highly connected in the transmission network, indicating that they may have more sexual partners. But unexpectedly, both a national online survey in China and our study found that MSM unwilling to screen for syphilis usually reported fewer sexual partners (35). These results suggested that the self-reported information from HIV-infected MSM may not be true. Therefore, we should interpret with caution the findings obtained through traditional epidemiological analysis according to the self-reported data. In contrast, the molecular epidemiological study was based on the HIV-1 sequences to construct the transmission networks. The results can be more objective and less likely to be affected by personal demographic information.

Our study is of great value for the precise prevention of a HIV-1 epidemic. (1) Our results further indicated that syphilis testing could be used as a proxy marker for the subgroup of MSM without syphilis testing during HIV-1 diagnosis. However, we would like to emphasize that syphilis testing *per se* was not associated with enhanced HIV-1 transmission and did not promote engagement of ART. As expected, we found that delayed diagnosis of HIV-1 and progressive disease stages were the key factors to affect the engagement of ART treatment. Furthermore, HIV-infected MSM without syphilis screening did not yield more from initiating ART earlier in our study. Consistent with the findings from previous studies, our study illustrated that the baseline CD4^+^ T cell count and early HIV-1 diagnosis were the major factors in predicting post-ART recovery of CD4^+^ cells (48), but not syphilis screening. (2) Molecular evolution and network analysis are important tools for the precise identification of HIV-1 transmission, which is usually difficult to define *via* traditional epidemiology surveys, due to the absence or inaccuracy of epidemiological data for HIV-infected individuals. (3) For the precise prevention and intervention of a HIV-1 epidemic, it is necessary to identify and distinguish the subgroups of HIV-infected subjects. We and others used a transmission network and cluster analysis to define potential non-disclosed MSM and their role in enhancing HIV-1 transmission.

Our study was subject to several limitations: (1) the study included 1 center, and was an observational study. Further investigations in multiple centers are needed to verify our findings. (2) The demographic information and the risk factors data such as the history of STIs, and the number of sexual partners in the past 6 months were self-reported although the information may not affect the phylogenetic analysis of HIV-1 sequences. (3) Syphilis screening data were captured from the HIV-1 primary care medical records only. If participants had gone elsewhere (i.e., hospital dermatology clinics) for syphilis screening, it would not be captured. (4) The construction of a HIV-1 transmission network based on only 1 HIV-1 pol gene may not be accurate enough and the data obtained were not authenticated using a wet lab procedure (20, 25).

## Conclusions

HIV-1 transmission network analysis revealed that HIV-infected MSM who refuse to undertake syphilis testing at the time of HIV-1 diagnosis was a specific subgroup of MSM and played an important role in HIV-1 transmission. Therefore, syphilis testing may be a proxy marker for identifying the specific sub-population in HIV-1 transmission. Specific prevention and intervention targeting MSM without syphilis testing are urgently needed. Our results could contribute to evidence-based policy making for the precise intervention of the HIV-1 epidemic.

## Data Availability Statement

The datasets presented in this study can be found in online repositories. The names of the repository/repositories and accession number(s) can be found in the article/Supplementary Materials.

## Ethics Statement

This study was approved by the Institutional Review Board of Guangzhou CDC (No. 2017030). The patients/participants provided their written informed consent to participate in this study.

## Author Contributions

ST and ZH conceived the study. HW, YL, QL and LH collected the data. LH and HY analyzed and interpreted the data. LH and ST wrote the manuscript. All authors reviewed, revised, and approved the final manuscript.

## Conflict of Interest

The authors declare that the research was conducted in the absence of any commercial or financial relationships that could be construed as a potential conflict of interest.

## Supplementary Material

Supplementary Figure 1 The flow chat of the study.

Supplementary Figure 2 The distribution of HIV-infected MSM with or without syphilis testing across and within the clusters of various sizes from 2008 to 2012 in Guangzhou, China. The HIV-1 transmission clusters identified by HIV-TRACE for MSM with or without syphilis testing during 2008–2012. The colors and shapes represent different groups of the HIV-1 sequences from the MSM with (green circle) or without (red square) syphilis testing and the HIV-1 reference sequences (light yellow triangle). The left and right y-axis are the total number of HIV-1 transmission clusters identified in this study and the proportion of these clusters, respectively. The x-axis shows the size of HIV-1 transmission clusters. The dusty blue bar represents the number of clusters while the lines indicate the clusters containing at least one HIV-infected MSM with (green) or without (orange) syphilis testing, respectively. The chart shows the coverage of syphilis testing among HIV-1 infected MSM clustered in 4 different cluster sizes. The blue and gray bar represent the proportion of HIV-1 infected MSM with and without syphilis testing in the corresponding clusters, respectively. MSM, men who have sex with men; HIV, human immunodeficiency virus.

Supplementary Figure 3 Phylogenetic transmission clusters for HIV-1 infected MSM in Guangzhou, China during 2008 and 2012. Phylogenetic HIV-1 transmission clusters identified by Cluster Picker. The outside circle displayed HIV-1 genotypes with different colors, i.e., HIV-1 CRF55_01B (yellow), CRF 01_AE (blue), CRF07_BC (green), subtype B (purple), and other HIV-1 genotypes (gray). The inside circle showed 46 transmission clusters with colored strips while the non-clustered sequences are displayed as white.

Supplementary Figure 4 Forest plot of the hazard ratio for the factors associated with antiretroviral initiation according to the disclosure of STIs history from 2008 to 2012 in Guangzhou, China. The forest plots summarize the hazard ratios (black squares) and their 95% confidential intervals (Cis, horizontal lines), as well as *P*-values for the interaction between ART initiation and the subgroup variables. A *P*-value < 0.05 is statistically significant. **P* < 0.05; ***P* < 0.01; ****P* < 0.001. MSM, men who have sex with men; STI, sexually transmitted infection; HIV, human immunodeficiency virus; AIDS, acquired immune deficiency syndrome.

Supplementary Table 1 Baseline characteristics of propensity-matched HIV-infected MSM diagnosed from 2008 to 2012 in Guangzhou, China.

Supplementary Table 2 Different percentage of STIs history between MSM with or without syphilis testing from 2008 to 2012 in Guangzhou, China.

Supplementary Table 3 Characteristics of HIV-infected MSM with or without antiviral treatment during 2008 and 2012 in Guangzhou, China.

Supplementary Table 4 Differences between ART-treated MSM with or without CD4^+^ T Cell recovery during 2008 and 2012 in Guangzhou, China.
