# Supplementary figures and images for: Syphilis Testing as a Proxy Marker for a Subgroup of Men Who Have Sex With Men With a Central Role in HIV-1 Transmission in Guangzhou, China
Source: Front Med (Lausanne). 2021 Jul 7;8:662689. doi: 10.3389/fmed.2021.662689 (PMC8293274; doi:10.3389/fmed.2021.662689)

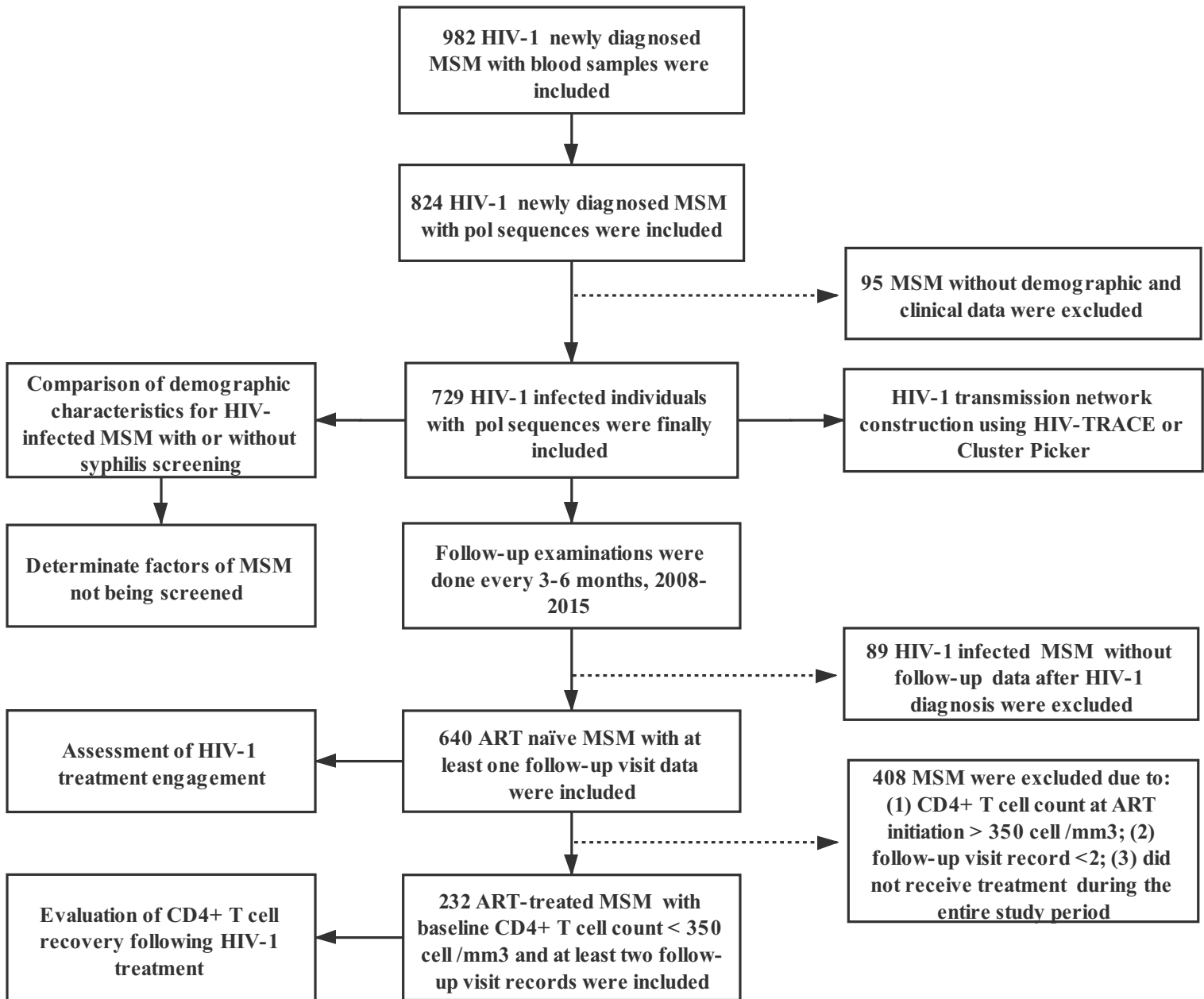

**Flow chart of the study**

Supplement: Supplementary Figure 1 — The flow chat of the study. [file Image_1.PDF]

**A**

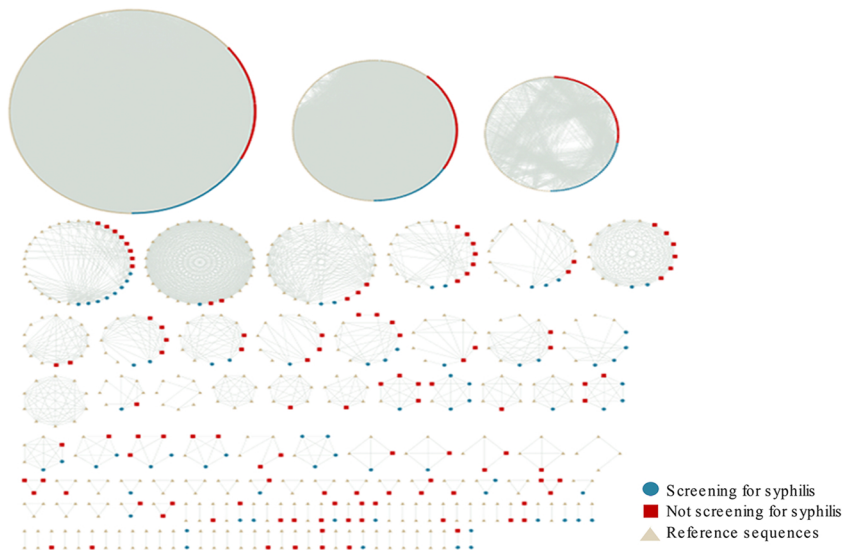

**B**

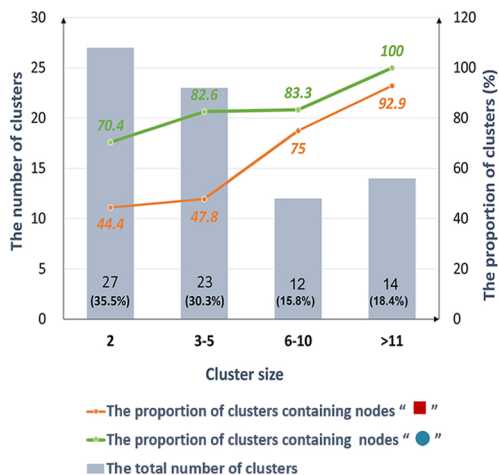

**C**

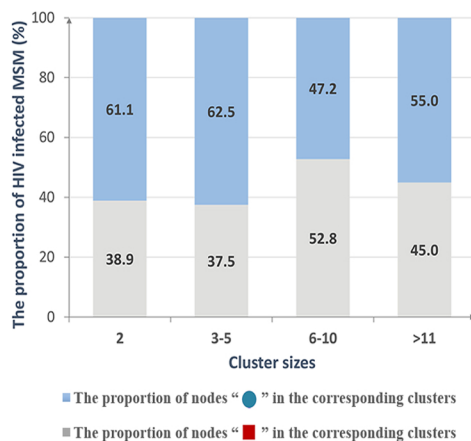

Supplement: Supplementary Figure 2 — The distribution of HIV-infected MSM with or without syphilis testing across and within the clusters of various sizes from 2008 to 2012 in Guangzhou, China. The HIV-1 transmission clusters identified by HIV-TRACE for MSM with or without syphilis testing during 2008–2012. The colors and shapes represent different groups of the HIV-1 sequences from the MSM with (green circle) or without (red square) syphilis testing and the HIV-1 reference sequences (light yellow triangle). The left and right y-axis are the total number of HIV-1 transmission clusters identified in this study and the proportion of these clusters, respectively. The x-axis shows the size of HIV-1 transmission clusters. The dusty blue bar represents the number of clusters while the lines indicate the clusters containing at least one HIV-infected MSM with (green) or without (orange) syphilis testing, respectively. The chart shows the coverage of syphilis testing among HIV-1 infected MSM clustered in 4 different cluster sizes. The blue and gray bar represent the proportion of HIV-1 infected MSM with and without syphilis testing in the corresponding clusters, respectively. MSM, men who have sex with men; HIV, human immunodeficiency virus. [file Image_2.PDF]

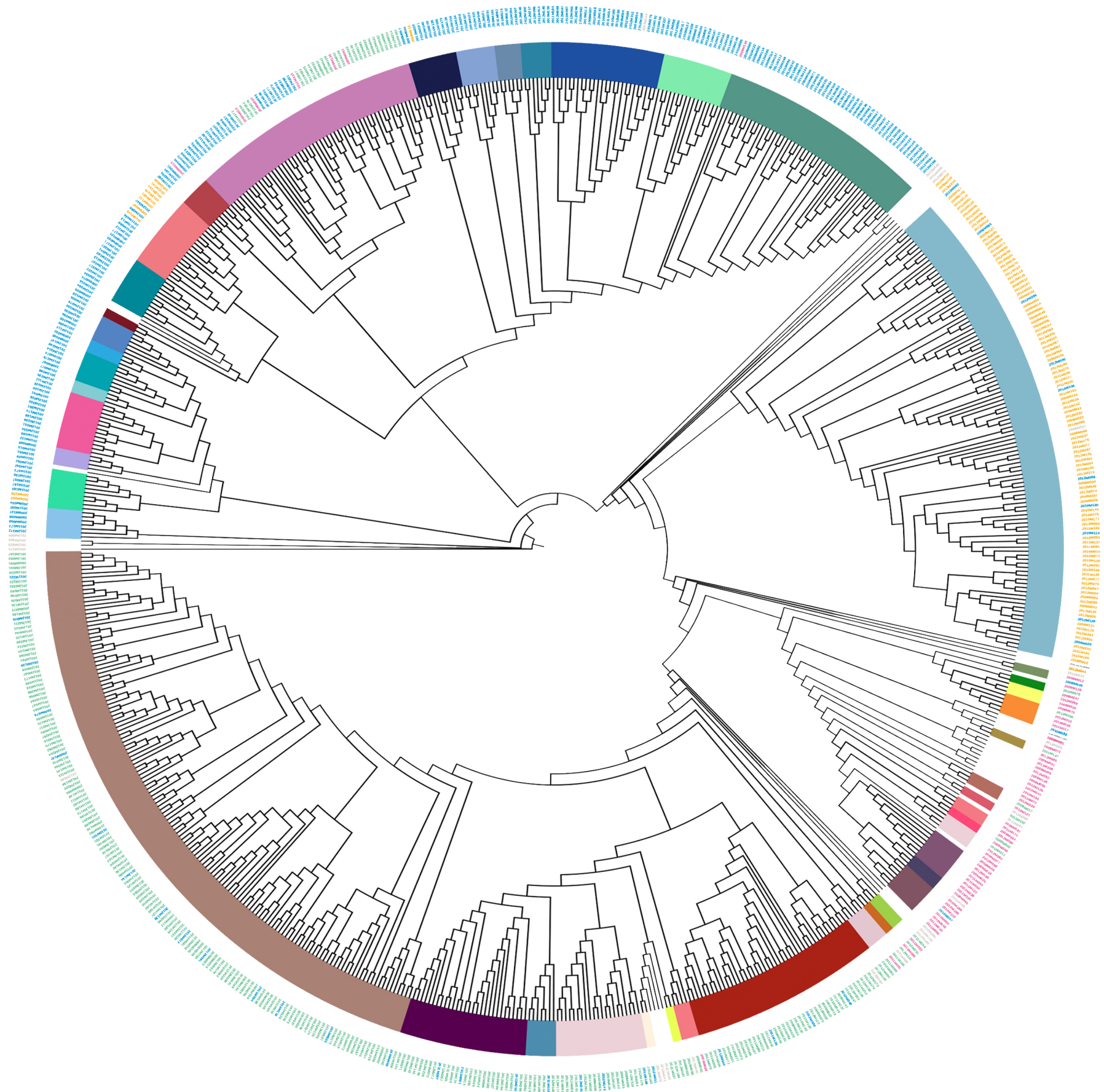

Supplement: Supplementary Figure 3 — Phylogenetic transmission clusters for HIV-1 infected MSM in Guangzhou, China during 2008 and 2012. Phylogenetic HIV-1 transmission clusters identified by Cluster Picker. The outside circle displayed HIV-1 genotypes with different colors, i.e., HIV-1 CRF55_01B (yellow), CRF 01_AE (blue), CRF07_BC (green), subtype B (purple), and other HIV-1 genotypes (gray). The inside circle showed 46 transmission clusters with colored strips while the non-clustered sequences are displayed as white. [file Image_3.PDF]

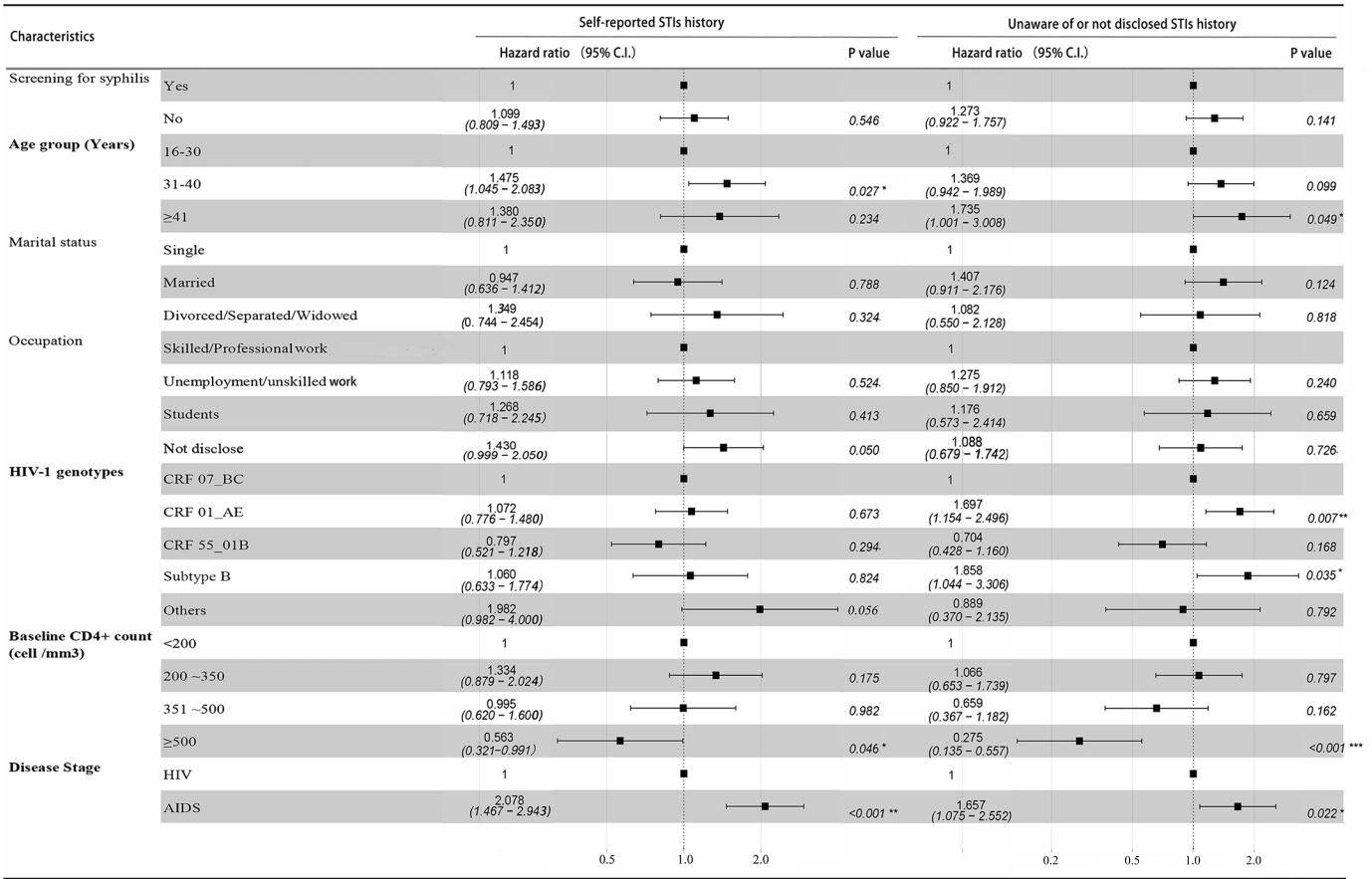

Supplement: Supplementary Figure 4 — Forest plot of the hazard ratio for the factors associated with antiretroviral initiation according to the disclosure of STIs history from 2008 to 2012 in Guangzhou, China. The forest plots summarize the hazard ratios (black squares) and their 95% confidential intervals (Cis, horizontal lines), as well as P-values for the interaction between ART initiation and the subgroup variables. A P-value < 0.05 is statistically significant. *P < 0.05; **P < 0.01; ***P < 0.001. MSM, men who have sex with men; STI, sexually transmitted infection; HIV, human immunodeficiency virus; AIDS, acquired immune deficiency syndrome. [file Image_4.PDF]
