# Supplementary material for: Syphilis Testing as a Proxy Marker for a Subgroup of Men Who Have Sex With Men With a Central Role in HIV-1 Transmission in Guangzhou, China
Source: Front Med (Lausanne). 2021 Jul 7;8:662689. doi: 10.3389/fmed.2021.662689 (PMC8293274; doi:10.3389/fmed.2021.662689)
Supplement: Supplementary Table 1 — Baseline characteristics of propensity-matched HIV-infected MSM diagnosed from 2008 to 2012 in Guangzhou, China. [file Data_Sheet_2.docx]

**Table S1. Baseline characteristics of propensity-matched HIV-infected MSM diagnosed from 2008 to 2012 in Guangzhou, China**

| **Characteristics** | | **Total**  **(n=440)** | **Syphilis screening (n, %)** | | **P value^#^** |
| --- | --- | --- | --- | --- | --- |
|  |  |  | **Yes (n=220)** | **No (n=220)** |  |
| Age group (Years) | 16-30 | 258 (58.6) | 135 (61.4) | 123 (55.9) | 0.500 |
|  | 31-40 | 134 (30.5) | 62 (28.2) | 72 (32.7) |  |
|  | ≥41 | 48 (10.9) | 23 (10.5) | 25 (11.4) |  |
| Marital status | Single | 310 (70.5) | 156 (70.9) | 154 (70.0) | 0.397 |
|  | Married | 99 (22.5) | 52 (23.6) | 47 (21.4) |  |
|  | Divorced/Separated/Widowed | 31 (7.0) | 12 (5.5) | 19 (8.6) |  |
| Education level | Lower than Senior high school | 103 (23.4) | 54 (24.5) | 49 (22.3) | 0.806 |
|  | Senior high school | 139 (31.6) | 70 (31.8) | 69 (31.4) |  |
|  | Junior college or higher | 198 (45.0) | 96 (43.6) | 102 (46.4) |  |
| Occupation | Skilled/professional work or students | 222 (50.5) | 112 (50.9) | 110 (50.0) | 0.849 |
|  | Unemployment, unskilled work or not disclose | 218 (49.5) | 108 (49.1) | 110 (50.0) |  |
| Number of sexual partners in the past 6 months | ≤ 1 | 79 (18.0) | 35 (15.9) | 44 (20.0) | 0.089 |
|  | 2~5 | 237 (53.9) | 130 (59.1) | 107 (48.6) |  |
|  | > 5 | 124 (28.2) | 55 (25.0) | 69 (31.4) |  |
| STIs history | Yes | 56 (12.7) | 30 (13.6) | 26 (11.8) | 0.380 |
|  | No | 164 (37.3) | 75 (34.1) | 89 (40.5) |  |
|  | Unknown | 220 (50.0) | 115 (52.3) | 105 (47.7) |  |
| HIV-1 genotype | CRF 07_BC | 171 (38.9) | 88 (40.0) | 83 (37.7) | 0.610 |
|  | CRF 01_AE | 165 (37.5) | 87 (39.5) | 78 (35.5) |  |
|  | CRF 55_01B | 55 (12.5) | 23 (10.5) | 32 (14.5) |  |
|  | Subtype B | 30 (6.8) | 13 (5.9) | 17 (7.7) |  |
|  | Others | 19 (4.3) | 9 (4.1) | 10 (4.5) |  |
| Baseline CD4+ counts (cell /mm3) | <200 | 64 (14.5) | 35 (15.9) | 29 (13.2) | 0.854 |
|  | ≤350 | 145 (33.0) | 73 (33.2) | 72 (32.7) |  |
|  | 351~500 | 140 (31.8) | 68 (30.9) | 72 (32.7) |  |
|  | >500 | 91 (20.7) | 44 (20.0) | 47 (21.4) |  |
| Disease Stage | HIV-1 infection | 294 (66.8) | 149 (67.7) | 145 (65.9) | 0.685 |
|  | AIDS | 146 (33.2) | 71 (32.3) | 75 (34.1) |  |

**^#^** Kruskal-wallis test or Chi-square tests, a p-value less than 0.05 is statistically significant. A p-value less than 0.05 is statistically significant.

Abbreviations: MSM, men who have sex with men; STI, sexually transmitted infection; HIV, human immunodeficiency virus; AIDS, acquired immune deficiency syndrome.

**Table S2. Different percentage of STIs history between MSM with or without syphilis testing from 2008 to 2012 in Guangzhou, China**

| **Characteristics** | | **Total** | **Syphilis screening (n, %)** | | **P value^*^** |
| --- | --- | --- | --- | --- | --- |
|  |  |  | **Yes** | **No** |  |
| STIs history | Yes | 101 | 35 (26.1) | 66 (27.7) | 0.127 |
|  | No | 271 | 99 (73.9) | 172 (72.3) |  |
| STIs history | Known^#^ | 372 | 134 (42.4) | 238 (57.6) | <0.001 |
|  | Unknown | 357 | 182 (57.6) | 175 (42.4) |  |

# MSM with and without self-reported history of STIs, STIs history including any history of hepatitis B virus, hepatitis C virus and syphilis infection

**^*^** Chi-square tests. A p-value less than 0.05 is statistically significant.

Abbreviations: MSM, men who have sex with men; STI, sexually transmitted infection;

**Table S3. Characteristics of HIV-infected MSM with or without antiviral treatment during 2008 and 2012 in Guangzhou, China**

| **Characteristics** | | **Total**  **(n=640)** | **Antiviral treatment (n, %)** | | **P value^#^** |
| --- | --- | --- | --- | --- | --- |
|  |  |  | **Yes (n=405)** | **No (n=235)** |  |
| Screening for syphilis | Yes | 281 | 156 (55.5) | 125 (44.5) | <0.001*** |
|  | No | 359 | 249 (69.4) | 110 (30.6) |  |
| Age group (Years) | 16-30 | 381 | 225 (59.1) | 156 (40.9) | <0.001*** |
|  | 31-40 | 187 | 125 (66.8) | 62 (33.2) |  |
|  | ≥41 | 72 | 55 (76.4) | 17 (23.6) |  |
| Marital status | Single | 458 | 274 (59.8) | 184 (40.2) | 0.017* |
|  | Married | 143 | 105 (73.4) | 38 (26.6) |  |
|  | Divorced/Separated/Widowed | 39 | 26 (66.7) | 13 (33.3) |  |
| Education level | Lower than Senior high school | 143 | 97 (67.8) | 46 (32.2) | 0.142 |
|  | Senior high school | 213 | 140 (65.7) | 73 (34.3) |  |
|  | Junior college or higher | 284 | 168 (59.2) | 116 (40.8) |  |
| Occupation | Skilled/Professional work | 377 | 225 (59.7) | 152 (40.3) | 0.064* |
|  | Students | 41 | 24 (58.5) | 17 (41.5) |  |
|  | Unemployment/unskilled work | 115 | 82 (71.3) | 33 (28.7) |  |
|  | Not disclose | 107 | 74 (69.2) | 33 (30.8) |  |
| Number of sexual partners in the past 6 months | ≤ 1 | 184 | 120 (65.2) | 64 (34.8) | 0.279 |
|  | 2~5 | 273 | 178 (65.2) | 95 (34.8) |  |
|  | > 5 | 183 | 107 (58.5) | 76 (41.5) |  |
| STIs history | Known | 329 | 233 (70.8) | 96 (29.2) | 0.001*** |
|  | Unknown | 311 | 172 (55.3) | 139(44.7) |  |
| HIV-1 genotype | CRF 07_BC | 238 | 134 (56.3) | 104 (43.7) | <0.001*** |
|  | CRF 01_AE | 232 | 160 (69.0) | 72 (31.0) |  |
|  | CRF 55_01B | 103 | 60 (58.3) | 43 (41.7) |  |
|  | Subtype B | 44 | 36 (81.8) | 8 (18.2) |  |
|  | Others | 23 | 15 (65.2) | 8 (34.8) |  |
| Baseline CD4+ counts (cell /mm^3^) | <200 | 88 | 78 (88.6) | 10 (11.4) | <0.001*** |
|  | 200 ~350 | 198 | 151 (76.3) | 47 (23.7) |  |
|  | 350 ~500 | 212 | 126 (59.4) | 86 (40.6) |  |
|  | ≥500 | 142 | 50 (35.2) | 92 (64.8) |  |
| Disease Stage | HIV-1 infection | 445 | 227 (51.0) | 218 (49.0) | <0.001*** |
|  | AIDS | 195 | 178 (91.3) | 17 (8.7) |  |

**^#^** Kruskal-wallis test or Chi-square tests.

A p-value less than 0.05 is statistically significant. *P < 0.05; **P < 0.01; ***P <0 .001.

Abbreviations: MSM, men who have sex with men; STI, sexually transmitted infection; HIV, human immunodeficiency virus; AIDS, acquired immune deficiency syndrome.

**Table S4. Differences between ART-treated MSM with or without CD4+ T Cell recovery during 2008 and 2012 in Guangzhou, China**

| **Characteristics** | | **Total** | **CD4+ count recovery (n, %)** | | **P value^#^** |
| --- | --- | --- | --- | --- | --- |
|  |  | **(n=232)** | **Yes (n=181)** | **No (n=51)** |  |
| Screening for syphilis | Yes | 92 | 78 (84.8) | 14 (15.2) | 0.044* |
|  | No | 140 | ­­­103 (73.6) | 37 (26.4) |  |
| Age group (Years) | 16-30 | 132 | 103 (78.0) | 29 (22.0) | 0.443 |
|  | 31-40 | 71 | 54 (76.1) | 17 (23.9) |  |
|  | ≥41 | 29 | 24 (82.8) | 5 (17.2) |  |
| Marital status | Single | 161 | 125 (77.6) | 36 (22.4) | 0.285 |
|  | Married | 55 | 46 (83.6) | 9 (16.4) |  |
|  | Divorced/Separated/Widowed | 16 | 10 (62.5) | 6 (37.5) |  |
| Education level | Lower than Senior high school | 52 | 38 (73.1) | 14 (26.9) | 0.572 |
|  | Senior high school | 63 | 51 (81.0) | 12 (19.0) |  |
|  | Junior college or higher | 117 | 92 (78.6) | 25 (21.4) |  |
| Occupation | Skilled/Professional work | 128 | 99 (77.3) | 29 (22.7) | 0.489 |
|  | Not disclose | 39 | 29 (74.4) | 10 (25.6) |  |
|  | Unemployment/Unskilled work | 44 | 35 (79.5) | 9 (20.5) |  |
|  | Students | 21 | 18 (85.7) | 3 (14.3) |  |
| Number of sexual partners in the past 6 months | ≤ 1 | 68 | 54 (79.4) | 14 (20.6) | 0.503 |
|  | 2~5 | 108 | 89 (82.4) | 19 (17.6) |  |
|  | > 5 | 56 | 38 (67.9) | 18 (32.1) |  |
| STIs history | Known | 141 | 108 (76.6) | 33 (23.4) | 0.950 |
|  | Unknown | 91 | 73 (80.2) | 18 (19.8) |  |
| HIV-1 genotype | CRF 07_BC | 86 | 76 (88.4) | 10 (11.6) | 0.001** |
|  | CRF 01_AE | 118 | 80 (67.8) | 38 (32.2) |  |
|  | Subtype B | 28 | 25 (89.3) | 3 (10.7) |  |
| CD4+ counts at HIV diagnosis (cell /mm^3^) | <200 | 59 | 27 (45.8.) | 32 (54.2) | <0.001*** |
|  | 200 ~350 | 113 | 96 (85.0) | 17 (15.0) |  |
|  | >350 | 60 | 58 (98.0) | 2 (2.0) |  |
| CD4+ counts at ART initiation (cell /mm3) | <200 | 88 | 44 (50.0) | 44 (50.0) | <0.001*** |
|  | 200 ~350 | 144 | 137 (95.1) | 7 (4.9) |  |
| Disease Stage | HIV-1 infection | 110 | 104 (94.5) | 6 (5.5) | <0.001*** |
|  | AIDS | 122 | 77 (63.1) | 45 (36.9) |  |
| Treatment regimen^&^ | NVP+3TC+AZT | 42 | 38 (21.0) | 4 (7.8) | 0.001** |
|  | EFV+3TC+AZT | 46 | 39 (21.5) | 7 (13.7) |  |
|  | EFV+3TC+TDF | 78 | 49 (27.1) | 29 (56.9) |  |
|  | Others | 66 | 55 (30.4) | 11 (21.6) |  |

**^#^** Kruskal-wallis test or Chi-square tests.

^&^ Adherence to the manual for China’s national free antiretroviral Therapy. 2nd ed. Beijing, China: People’s Medical Publishing House 2008.

A p-value less than 0.05 is statistically significant. *P < 0.05; **P < 0.01; ***P <0 .001.

Abbreviations: MSM, men who have sex with men; STI, sexually transmitted infection; HIV, human immunodeficiency virus; AIDS, acquired immune deficiency syndrome; ART, antiretroviral therapy; 3TC, Lamivudine; AZT, Zidovudine; TDF, Tenofovir; EFV, Efavirenz.
