# Supplementary material for: Syphilis Testing as a Proxy Marker for a Subgroup of Men Who Have Sex With Men With a Central Role in HIV-1 Transmission in Guangzhou, China
Source: Front Med (Lausanne). 2021 Jul 7;8:662689. doi: 10.3389/fmed.2021.662689 (PMC8293274; doi:10.3389/fmed.2021.662689)
Supplement: Supplementary file 6 [file Data_Sheet_1.DOCX]

MN424585 BankIt2261090 B.GZ.2008M013_2008.63

MN424783 BankIt2261090 CRF01_AE.GZ.2008M045_2008.08

MN424589 BankIt2261090 B.GZ.2008M047_2008.13

MN424590 BankIt2261090 B.GZ.2008M048_2008.21

MN424591 BankIt2261090 B.GZ.2008M061_2008.76

MN424593 BankIt2261090 B.GZ.2009M005_2009.37

MN424595 BankIt2261090 B.GZ.2009M010_2009.18

MN424597 BankIt2261090 B.GZ.2009M027_2009.48

MN424598 BankIt2261090 B.GZ.2009M028_2009.49

MN424806 BankIt2261090 CRF01_AE.GZ.2009M029_2009.5

MN424599 BankIt2261090 B.GZ.2009M036_2009.55

MN424600 BankIt2261090 B.GZ.2009M041_2009.64

MN424605 BankIt2261090 B.GZ.2009M077_2009.44

MN424606 BankIt2261090 B.GZ.2009M085_2009.54

MN427026 BankIt2261423 CRF55_01B.GZ.2009M100_2009.63

MN424828 BankIt2261090 CRF01_AE.GZ.2009M101_2009.64

MN424608 BankIt2261090 B.GZ.2009M119_2009.79

MN424609 BankIt2261090 B.GZ.2009M129_2009.86

MN424611 BankIt2261090 B.GZ.2009M138_2009.93

MN424612 BankIt2261090 B.GZ.2009M145_2009.98

MN427043 BankIt2261423 CRF55_01B.GZ.2010M010_2010.95

MN424850 BankIt2261090 CRF01_AE.GZ.2010M023_2010.93

MN424865 BankIt2261090 CRF01_AE.GZ.2010M077_2010.41

MN424616 BankIt2261090 B.GZ.2010M089_2010.05

MN424868 BankIt2261090 CRF01_AE.GZ.2010M092_2010.08

MN424883 BankIt2261090 CRF01_AE.GZ.2010M128_2010.53

MN424888 BankIt2261090 CRF01_AE.GZ.2010M137_2010.59

MN424892 BankIt2261090 CRF01_AE.GZ.2010M148_2010.7

MN424620 BankIt2261090 B.GZ.2010M155_2010.74

MN424909 BankIt2261090 CRF01_AE.GZ.2011M004_2011.02

MN424914 BankIt2261090 CRF01_AE.GZ.2011M016_2011.19

MN424624 BankIt2261090 B.GZ.2011M031_2011.36

MN424625 BankIt2261090 B.GZ.2011M033_2011.37

MN426040 BankIt2261420 CRF07_BC.GZ.2011M037_2011.41

MN424626 BankIt2261090 B.GZ.2011M040_2011.43

MN424920 BankIt2261090 CRF01_AE.GZ.2011M043_2011.45

MN424925 BankIt2261090 CRF01_AE.GZ.2011M056_2011.51

MN424629 BankIt2261090 B.GZ.2011M072_2011.64

MN424938 BankIt2261090 CRF01_AE.GZ.2011M098_2011.81

MN424963 BankIt2261090 CRF01_AE.GZ.2011M185_2011.67

MN424970 BankIt2261090 CRF01_AE.GZ.2011M204_2011.87

MN424976 BankIt2261090 CRF01_AE.GZ.2011M223_2011.96

MN424985 BankIt2261090 CRF01_AE.GZ.2012M006_2012.04

MN424988 BankIt2261090 CRF01_AE.GZ.2012M010_2012.12

MN424993 BankIt2261090 CRF01_AE.GZ.2012M028_2012.27

MN425001 BankIt2261090 CRF01_AE.GZ.2012M042_2012.35

MN424637 BankIt2261090 B.GZ.2012M044_2012.38

MN425003 BankIt2261090 CRF01_AE.GZ.2012M048_2012.39

MN424638 BankIt2261090 B.GZ.2012M066_2012.58

MN424639 BankIt2261090 B.GZ.2012M082_2012.72

MN424640 BankIt2261090 B.GZ.2012M085_2012.78

MN427103 BankIt2261425 CRF55_01B.GZ.2012M092_2012.8

MN425016 BankIt2261090 CRF01_AE.GZ.2012M094_2012.81

MN425017 BankIt2261090 CRF01_AE.GZ.2012M096_2012.81

MN425024 BankIt2261090 CRF01_AE.GZ.2012M112_2012.89

MN424641 BankIt2261090 B.GZ.2012M126_2012.96

MN425029 BankIt2261090 CRF01_AE.GZ.2012M129_2012.97

MN426175 BankIt2261422 CRF07_BC.GZ.2012M140_2012.12

MN425032 BankIt2261090 CRF01_AE.GZ.2012M143_2012.12

MN425036 BankIt2261090 CRF01_AE.GZ.2012M148_2012.13

MN424642 BankIt2261090 B.GZ.2012M190_2012.23

MN424643 BankIt2261090 B.GZ.2012M199_2012.28

MN425051 BankIt2261090 CRF01_AE.GZ.2012M205_2012.29

MN425062 BankIt2261090 CRF01_AE.GZ.2012M230_2012.36

MN424645 BankIt2261090 B.GZ.2012M252_2012.43

MN425071 BankIt2261090 CRF01_AE.GZ.2012M258_2012.45

MN425073 BankIt2261090 CRF01_AE.GZ.2012M278_2012.52

MN425078 BankIt2261090 CRF01_AE.GZ.2012M292_2012.57

MN425082 BankIt2261090 CRF01_AE.GZ.2012M300_2012.61

MN425088 BankIt2261417 CRF01_AE.GZ.2012M317_2012.6

MN425090 BankIt2261417 CRF01_AE.GZ.2012M324_2012.63

MN425092 BankIt2261417 CRF01_AE.GZ.2012M334_2012.68

MN425094 BankIt2261417 CRF01_AE.GZ.2012M338_2012.71

MN425096 BankIt2261417 CRF01_AE.GZ.2012M344_2012.72

MN424785 BankIt2261090 CRF01_AE.GZ.2008M056_2008.51

MN424786 BankIt2261090 CRF01_AE.GZ.2008M057_2008.51

MN424789 BankIt2261090 CRF01_AE.GZ.2008M069_2008.96

MN424790 BankIt2261090 CRF01_AE.GZ.2008M071_2008.96

MN424795 BankIt2261090 CRF01_AE.GZ.2008M082_2009.05

MN424821 BankIt2261090 CRF01_AE.GZ.2009M075_2009.44

MN424826 BankIt2261090 CRF01_AE.GZ.2009M096_2009.61

MN424830 BankIt2261090 CRF01_AE.GZ.2009M114_2009.73

MN424831 BankIt2261090 CRF01_AE.GZ.2009M118_2009.79

MN424832 BankIt2261090 CRF01_AE.GZ.2009M120_2009.81

MN424867 BankIt2261090 CRF01_AE.GZ.2010M090_2010.05

MN424873 BankIt2261090 CRF01_AE.GZ.2010M103_2010.3

MN424874 BankIt2261090 CRF01_AE.GZ.2010M107_2010.32

MN424885 BankIt2261090 CRF01_AE.GZ.2010M132_2010.58

MN424886 BankIt2261090 CRF01_AE.GZ.2010M134_2010.59

MN424897 BankIt2261090 CRF01_AE.GZ.2010M160_2010.81

MN424900 BankIt2261090 CRF01_AE.GZ.2010M177_2010.99

MN424910 BankIt2261090 CRF01_AE.GZ.2011M005_2011.03

MN424916 BankIt2261090 CRF01_AE.GZ.2011M029_2011.32

MN424921 BankIt2261090 CRF01_AE.GZ.2011M049_2011.48

MN424923 BankIt2261090 CRF01_AE.GZ.2011M054_2011.51

MN424924 BankIt2261090 CRF01_AE.GZ.2011M055_2011.51

MN424929 BankIt2261090 CRF01_AE.GZ.2011M068_2011.61

MN424937 BankIt2261090 CRF01_AE.GZ.2011M097_2011.8

MN424944 BankIt2261090 CRF01_AE.GZ.2011M122_2011.97

MN424987 BankIt2261090 CRF01_AE.GZ.2012M009_2012.12

MN424990 BankIt2261090 CRF01_AE.GZ.2012M013_2012.14

MN424992 BankIt2261090 CRF01_AE.GZ.2012M025_2012.25

MN425000 BankIt2261090 CRF01_AE.GZ.2012M040_2012.34

MN425002 BankIt2261090 CRF01_AE.GZ.2012M043_2012.37

MN425004 BankIt2261090 CRF01_AE.GZ.2012M051_2012.43

MN425014 BankIt2261090 CRF01_AE.GZ.2012M089_2012.79

MN425021 BankIt2261090 CRF01_AE.GZ.2012M101_2012.85

MN425023 BankIt2261090 CRF01_AE.GZ.2012M108_2012.87

MN425026 BankIt2261090 CRF01_AE.GZ.2012M114_2012.91

MN425027 BankIt2261090 CRF01_AE.GZ.2012M122_2012.95

MN425030 BankIt2261090 CRF01_AE.GZ.2012M133_2012.01

MN425031 BankIt2261090 CRF01_AE.GZ.2012M137_2012.11

MN425037 BankIt2261090 CRF01_AE.GZ.2012M154_2012.14

MN425038 BankIt2261090 CRF01_AE.GZ.2012M155_2012.14

MN425039 BankIt2261090 CRF01_AE.GZ.2012M159_2012.16

MN425042 BankIt2261090 CRF01_AE.GZ.2012M170_2012.17

MN425046 BankIt2261090 CRF01_AE.GZ.2012M183_2012.2

MN425048 BankIt2261090 CRF01_AE.GZ.2012M191_2012.3

MN425050 BankIt2261090 CRF01_AE.GZ.2012M198_2012.27

MN425053 BankIt2261090 CRF01_AE.GZ.2012M208_2012.31

MN424778 BankIt2261090 CRF01_AE.GZ.2008M021_2008.44

MN424779 BankIt2261090 CRF01_AE.GZ.2008M031_2008.93

MN424780 BankIt2261090 CRF01_AE.GZ.2008M037_2008.94

MN424781 BankIt2261090 CRF01_AE.GZ.2008M038_2008.96

MN424812 BankIt2261090 CRF01_AE.GZ.2009M044_2009.8

MN424813 BankIt2261090 CRF01_AE.GZ.2009M046_2009.88

MN424814 BankIt2261090 CRF01_AE.GZ.2009M047_2009.93

MN424848 BankIt2261090 CRF01_AE.GZ.2010M015_2010.47

MN424858 BankIt2261090 CRF01_AE.GZ.2010M058_2010.4

MN424860 BankIt2261090 CRF01_AE.GZ.2010M063_2010.61

MN424864 BankIt2261090 CRF01_AE.GZ.2010M076_2010.36

MN424945 BankIt2261090 CRF01_AE.GZ.2011M128_2011.04

MN424948 BankIt2261090 CRF01_AE.GZ.2011M135_2011.24

MN424952 BankIt2261090 CRF01_AE.GZ.2011M151_2011.34

MN424960 BankIt2261090 CRF01_AE.GZ.2011M179_2011.65

MN424973 BankIt2261090 CRF01_AE.GZ.2011M209_2011.9

MN424977 BankIt2261090 CRF01_AE.GZ.2011M227_2011.98

MN424978 BankIt2261090 CRF01_AE.GZ.2011M228_2011.98

MN425055 BankIt2261090 CRF01_AE.GZ.2012M215_2012.32

MN425056 BankIt2261090 CRF01_AE.GZ.2012M216_2012.32

MN425063 BankIt2261090 CRF01_AE.GZ.2012M234_2012.38

MN425072 BankIt2261090 CRF01_AE.GZ.2012M263_2012.46

MN425074 BankIt2261090 CRF01_AE.GZ.2012M283_2012.53

MN425077 BankIt2261090 CRF01_AE.GZ.2012M287_2012.55

MN425079 BankIt2261090 CRF01_AE.GZ.2012M293_2012.56

MN425080 BankIt2261090 CRF01_AE.GZ.2012M294_2012.56

MN425083 BankIt2261090 CRF01_AE.GZ.2012M301_2012.58

MN425085 BankIt2261417 CRF01_AE.GZ.2012M312_2012.6

MN425087 BankIt2261417 CRF01_AE.GZ.2012M315_2012.62

MN425091 BankIt2261417 CRF01_AE.GZ.2012M327_2012.64

MN425098 BankIt2261417 CRF01_AE.GZ.2012M348_2012.73

MN424782 BankIt2261090 CRF01_AE.GZ.2008M040_2008.96

MN424807 BankIt2261090 CRF01_AE.GZ.2009M031_2009.5

MN424947 BankIt2261090 CRF01_AE.GZ.2011M134_2011.25

MN424950 BankIt2261090 CRF01_AE.GZ.2011M139_2011.3

MN424965 BankIt2261090 CRF01_AE.GZ.2011M192_2011.77

MN424967 BankIt2261090 CRF01_AE.GZ.2011M200_2011.85

MN424974 BankIt2261090 CRF01_AE.GZ.2011M212_2011.92

MN425059 BankIt2261090 CRF01_AE.GZ.2012M223_2012.34

MN425880 BankIt2261420 CRF07_BC.GZ.2008M011_2008.62

MN425881 BankIt2261420 CRF07_BC.GZ.2008M016_2008.83

MN425891 BankIt2261420 CRF07_BC.GZ.2008M046_2008.13

MN425894 BankIt2261420 CRF07_BC.GZ.2008M065_2008.92

MN425895 BankIt2261420 CRF07_BC.GZ.2008M068_2008.95

MN425896 BankIt2261420 CRF07_BC.GZ.2008M070_2008.37

MN425897 BankIt2261420 CRF07_BC.GZ.2008M073_2009

MN425898 BankIt2261420 CRF07_BC.GZ.2008M081_2008.35

MN425928 BankIt2261420 CRF07_BC.GZ.2009M060_2009.22

MN425931 BankIt2261420 CRF07_BC.GZ.2009M091_2009.57

MN425932 BankIt2261420 CRF07_BC.GZ.2009M092_2009.57

MN425933 BankIt2261420 CRF07_BC.GZ.2009M099_2009.61

MN425934 BankIt2261420 CRF07_BC.GZ.2009M112_2009.71

MN425935 BankIt2261420 CRF07_BC.GZ.2009M124_2009.82

MN425937 BankIt2261420 CRF07_BC.GZ.2009M139_2009.93

MN425938 BankIt2261420 CRF07_BC.GZ.2009M142_2009.94

MN425984 BankIt2261420 CRF07_BC.GZ.2010M083_2010.01

MN425985 BankIt2261420 CRF07_BC.GZ.2010M088_2010.04

MN425986 BankIt2261420 CRF07_BC.GZ.2010M091_2010.07

MN425987 BankIt2261420 CRF07_BC.GZ.2010M095_2010.21

MN425988 BankIt2261420 CRF07_BC.GZ.2010M098_2010.23

MN425990 BankIt2261420 CRF07_BC.GZ.2010M108_2010.36

MN425991 BankIt2261420 CRF07_BC.GZ.2010M109_2010.36

MN425992 BankIt2261420 CRF07_BC.GZ.2010M124_2010.49

MN425993 BankIt2261420 CRF07_BC.GZ.2010M127_2010.51

MN425994 BankIt2261420 CRF07_BC.GZ.2010M135_2010.59

MN425995 BankIt2261420 CRF07_BC.GZ.2010M138_2010.6

MN425996 BankIt2261420 CRF07_BC.GZ.2010M140_2010.61

MN425997 BankIt2261420 CRF07_BC.GZ.2010M142_2010.64

MN425998 BankIt2261420 CRF07_BC.GZ.2010M151_2010.73

MN426000 BankIt2261420 CRF07_BC.GZ.2010M161_2010.84

MN426001 BankIt2261420 CRF07_BC.GZ.2010M162_2010.84

MN426002 BankIt2261420 CRF07_BC.GZ.2010M163_2010.88

MN426003 BankIt2261420 CRF07_BC.GZ.2010M170_2010.94

MN426005 BankIt2261420 CRF07_BC.GZ.2010M173_2010.96

MN426006 BankIt2261420 CRF07_BC.GZ.2010M175_2010.97

MN426007 BankIt2261420 CRF07_BC.GZ.2010M176_2010.97

MN426029 BankIt2261420 CRF07_BC.GZ.2011M009_2011.05

MN426030 BankIt2261420 CRF07_BC.GZ.2011M012_2011.13

MN426032 BankIt2261420 CRF07_BC.GZ.2011M015_2011.17

MN426033 BankIt2261420 CRF07_BC.GZ.2011M018_2011.21

MN426034 BankIt2261420 CRF07_BC.GZ.2011M019_2011.23

MN426035 BankIt2261420 CRF07_BC.GZ.2011M022_2011.25

MN426036 BankIt2261420 CRF07_BC.GZ.2011M025_2011.27

MN426037 BankIt2261420 CRF07_BC.GZ.2011M026_2011.28

MN426038 BankIt2261420 CRF07_BC.GZ.2011M027_2011.28

MN426039 BankIt2261420 CRF07_BC.GZ.2011M034_2011.38

MN426042 BankIt2261420 CRF07_BC.GZ.2011M042_2011.43

MN426043 BankIt2261420 CRF07_BC.GZ.2011M046_2011.47

MN426044 BankIt2261420 CRF07_BC.GZ.2011M047_2011.47

MN426045 BankIt2261420 CRF07_BC.GZ.2011M048_2011.47

MN426046 BankIt2261420 CRF07_BC.GZ.2011M059_2011.55

MN426047 BankIt2261420 CRF07_BC.GZ.2011M060_2011.55

MN426048 BankIt2261420 CRF07_BC.GZ.2011M061_2011.55

MN426049 BankIt2261420 CRF07_BC.GZ.2011M063_2011.55

MN426050 BankIt2261420 CRF07_BC.GZ.2011M067_2011.61

MN426051 BankIt2261420 CRF07_BC.GZ.2011M069_2011.61

MN426052 BankIt2261420 CRF07_BC.GZ.2011M070_2011.64

MN426053 BankIt2261420 CRF07_BC.GZ.2011M080_2011.7

MN426054 BankIt2261420 CRF07_BC.GZ.2011M084_2011.73

MN426055 BankIt2261420 CRF07_BC.GZ.2011M087_2011.74

MN426056 BankIt2261420 CRF07_BC.GZ.2011M090_2011.78

MN426057 BankIt2261420 CRF07_BC.GZ.2011M091_2011.78

MN426058 BankIt2261420 CRF07_BC.GZ.2011M093_2011.8

MN426059 BankIt2261420 CRF07_BC.GZ.2011M094_2011.8

MN426060 BankIt2261420 CRF07_BC.GZ.2011M096_2011.8

MN426061 BankIt2261420 CRF07_BC.GZ.2011M099_2011.82

MN426062 BankIt2261420 CRF07_BC.GZ.2011M100_2011.82

MN426063 BankIt2261420 CRF07_BC.GZ.2011M101_2011.82

MN426064 BankIt2261420 CRF07_BC.GZ.2011M107_2011.85

MN426065 BankIt2261420 CRF07_BC.GZ.2011M108_2011.85

MN426066 BankIt2261420 CRF07_BC.GZ.2011M109_2011.87

MN426067 BankIt2261420 CRF07_BC.GZ.2011M118_2011.93

MN426131 BankIt2261422 CRF07_BC.GZ.2012M001_2012.01

MN426132 BankIt2261422 CRF07_BC.GZ.2012M002_2012.03

MN426133 BankIt2261422 CRF07_BC.GZ.2012M008_2012.12

MN426134 BankIt2261422 CRF07_BC.GZ.2012M015_2012.18

MN426135 BankIt2261422 CRF07_BC.GZ.2012M016_2012.21

MN426136 BankIt2261422 CRF07_BC.GZ.2012M019_2012.22

MN426137 BankIt2261422 CRF07_BC.GZ.2012M023_2012.24

MN426138 BankIt2261422 CRF07_BC.GZ.2012M024_2012.25

MN426139 BankIt2261422 CRF07_BC.GZ.2012M026_2012.26

MN426140 BankIt2261422 CRF07_BC.GZ.2012M027_2012.26

MN426141 BankIt2261422 CRF07_BC.GZ.2012M029_2012.28

MN426142 BankIt2261422 CRF07_BC.GZ.2012M032_2012.28

MN426143 BankIt2261422 CRF07_BC.GZ.2012M041_2012.35

MN426146 BankIt2261422 CRF07_BC.GZ.2012M053_2012.43

MN426147 BankIt2261422 CRF07_BC.GZ.2012M055_2012.49

MN426148 BankIt2261422 CRF07_BC.GZ.2012M058_2012.52

MN426149 BankIt2261422 CRF07_BC.GZ.2012M064_2012.55

MN426152 BankIt2261422 CRF07_BC.GZ.2012M088_2012.78

MN426153 BankIt2261422 CRF07_BC.GZ.2012M090_2012.8

MN426154 BankIt2261422 CRF07_BC.GZ.2012M093_2012.8

MN426155 BankIt2261422 CRF07_BC.GZ.2012M098_2012.83

MN426156 BankIt2261422 CRF07_BC.GZ.2012M103_2012.85

MN426157 BankIt2261422 CRF07_BC.GZ.2012M104_2012.85

MN426158 BankIt2261422 CRF07_BC.GZ.2012M106_2012.87

MN426159 BankIt2261422 CRF07_BC.GZ.2012M107_2012.87

MN426160 BankIt2261422 CRF07_BC.GZ.2012M109_2012.89

MN426161 BankIt2261422 CRF07_BC.GZ.2012M110_2012.89

MN426162 BankIt2261422 CRF07_BC.GZ.2012M115_2012.91

MN426163 BankIt2261422 CRF07_BC.GZ.2012M116_2012.91

MN426164 BankIt2261422 CRF07_BC.GZ.2012M117_2012.93

MN426165 BankIt2261422 CRF07_BC.GZ.2012M119_2012.93

MN426166 BankIt2261422 CRF07_BC.GZ.2012M121_2012.93

MN426167 BankIt2261422 CRF07_BC.GZ.2012M123_2012.95

MN426168 BankIt2261422 CRF07_BC.GZ.2012M124_2012.95

MN426169 BankIt2261422 CRF07_BC.GZ.2012M125_2012.96

MN426170 BankIt2261422 CRF07_BC.GZ.2012M131_2012.01

MN426171 BankIt2261422 CRF07_BC.GZ.2012M132_2012.01

MN426173 BankIt2261422 CRF07_BC.GZ.2012M136_2012.1

MN426176 BankIt2261422 CRF07_BC.GZ.2012M150_2012.14

MN426177 BankIt2261422 CRF07_BC.GZ.2012M158_2012.15

MN426178 BankIt2261422 CRF07_BC.GZ.2012M160_2012.15

MN426179 BankIt2261422 CRF07_BC.GZ.2012M162_2012.15

MN426180 BankIt2261422 CRF07_BC.GZ.2012M163_2012.15

MN426181 BankIt2261422 CRF07_BC.GZ.2012M166_2012.15

MN426182 BankIt2261422 CRF07_BC.GZ.2012M167_2012.16

MN426184 BankIt2261422 CRF07_BC.GZ.2012M173_2012.18

MN426185 BankIt2261422 CRF07_BC.GZ.2012M174_2012.18

MN426186 BankIt2261422 CRF07_BC.GZ.2012M175_2012.18

MN426187 BankIt2261422 CRF07_BC.GZ.2012M179_2012.19

MN426188 BankIt2261422 CRF07_BC.GZ.2012M186_2012.22

MN426189 BankIt2261422 CRF07_BC.GZ.2012M187_2012.22

MN426190 BankIt2261422 CRF07_BC.GZ.2012M189_2012.26

MN426191 BankIt2261422 CRF07_BC.GZ.2012M194_2012.26

MN426192 BankIt2261422 CRF07_BC.GZ.2012M195_2012.27

MN426193 BankIt2261422 CRF07_BC.GZ.2012M197_2012.27

MN426194 BankIt2261422 CRF07_BC.GZ.2012M200_2012.29

MN426195 BankIt2261422 CRF07_BC.GZ.2012M203_2012.29

MN426196 BankIt2261422 CRF07_BC.GZ.2012M206_2012.29

MN425889 BankIt2261420 CRF07_BC.GZ.2008M041_2008.43

MN425920 BankIt2261420 CRF07_BC.GZ.2009M003_2009.32

MN425921 BankIt2261420 CRF07_BC.GZ.2009M006_2009.41

MN425922 BankIt2261420 CRF07_BC.GZ.2009M019_2009.45

MN425923 BankIt2261420 CRF07_BC.GZ.2009M021_2009.45

MN425924 BankIt2261420 CRF07_BC.GZ.2009M024_2009.46

MN425925 BankIt2261420 CRF07_BC.GZ.2009M035_2009.47

MN425964 BankIt2261420 CRF07_BC.GZ.2010M018_2010.59

MN425968 BankIt2261420 CRF07_BC.GZ.2010M030_2010.87

MN425970 BankIt2261420 CRF07_BC.GZ.2010M036_2010.77

MN425976 BankIt2261420 CRF07_BC.GZ.2010M057_2010.36

MN425977 BankIt2261420 CRF07_BC.GZ.2010M061_2010.49

MN425979 BankIt2261420 CRF07_BC.GZ.2010M072_2010.27

MN425980 BankIt2261420 CRF07_BC.GZ.2010M078_2010.42

MN425981 BankIt2261420 CRF07_BC.GZ.2010M079_2010.5

MN425982 BankIt2261420 CRF07_BC.GZ.2010M080_2010.58

MN426070 BankIt2261420 CRF07_BC.GZ.2011M124_2011.02

MN426072 BankIt2261420 CRF07_BC.GZ.2011M126_2011.02

MN426074 BankIt2261420 CRF07_BC.GZ.2011M129_2011.16

MN426075 BankIt2261420 CRF07_BC.GZ.2011M132_2011.24

MN426076 BankIt2261420 CRF07_BC.GZ.2011M133_2011.23

MN426077 BankIt2261420 CRF07_BC.GZ.2011M137_2011.28

MN426078 BankIt2261420 CRF07_BC.GZ.2011M140_2011.31

MN426083 BankIt2261420 CRF07_BC.GZ.2011M149_2011.33

MN426085 BankIt2261422 CRF07_BC.GZ.2011M155_2011.38

MN426087 BankIt2261422 CRF07_BC.GZ.2011M158_2011.37

MN426088 BankIt2261422 CRF07_BC.GZ.2011M159_2011.38

MN426089 BankIt2261422 CRF07_BC.GZ.2011M160_2011.39

MN426092 BankIt2261422 CRF07_BC.GZ.2011M164_2011.43

MN426093 BankIt2261422 CRF07_BC.GZ.2011M165_2011.45

MN426095 BankIt2261422 CRF07_BC.GZ.2011M172_2011.59

MN426096 BankIt2261422 CRF07_BC.GZ.2011M176_2011.61

MN426097 BankIt2261422 CRF07_BC.GZ.2011M178_2011.61

MN426099 BankIt2261422 CRF07_BC.GZ.2011M183_2011.67

MN426102 BankIt2261422 CRF07_BC.GZ.2011M189_2011.71

MN426104 BankIt2261422 CRF07_BC.GZ.2011M196_2011.79

MN426105 BankIt2261422 CRF07_BC.GZ.2011M202_2011.85

MN426106 BankIt2261422 CRF07_BC.GZ.2011M205_2011.88

MN426109 BankIt2261422 CRF07_BC.GZ.2011M216_2011.94

MN426113 BankIt2261422 CRF07_BC.GZ.2011M224_2011.97

MN426115 BankIt2261422 CRF07_BC.GZ.2011M226_2011.97

MN426205 BankIt2261422 CRF07_BC.GZ.2012M226_2012.35

MN426208 BankIt2261422 CRF07_BC.GZ.2012M236_2012.4

MN426209 BankIt2261422 CRF07_BC.GZ.2012M240_2012.41

MN426212 BankIt2261422 CRF07_BC.GZ.2012M248_2012.43

MN426214 BankIt2261422 CRF07_BC.GZ.2012M254_2012.44

MN426215 BankIt2261422 CRF07_BC.GZ.2012M257_2012.45

MN426216 BankIt2261422 CRF07_BC.GZ.2012M261_2012.45

MN426222 BankIt2261422 CRF07_BC.GZ.2012M272_2012.5

MN426223 BankIt2261422 CRF07_BC.GZ.2012M276_2012.51

MN426224 BankIt2261422 CRF07_BC.GZ.2012M277_2012.51

MN426225 BankIt2261422 CRF07_BC.GZ.2012M279_2012.52

MN426226 BankIt2261422 CRF07_BC.GZ.2012M282_2012.53

MN426227 BankIt2261422 CRF07_BC.GZ.2012M284_2012.53

MN426228 BankIt2261422 CRF07_BC.GZ.2012M288_2012.55

MN426230 BankIt2261422 CRF07_BC.GZ.2012M290_2012.55

MN426231 BankIt2261422 CRF07_BC.GZ.2012M291_2012.55

MN426232 BankIt2261422 CRF07_BC.GZ.2012M296_2012.56

MN426233 BankIt2261422 CRF07_BC.GZ.2012M298_2012.58

MN426235 BankIt2261422 CRF07_BC.GZ.2012M306_2012.59

MN426236 BankIt2261422 CRF07_BC.GZ.2012M308_2012.59

MN426238 BankIt2261422 CRF07_BC.GZ.2012M311_2012.6

MN426239 BankIt2261422 CRF07_BC.GZ.2012M318_2012.69

MN426240 BankIt2261422 CRF07_BC.GZ.2012M321_2012.62

MN426241 BankIt2261422 CRF07_BC.GZ.2012M322_2012.62

MN426242 BankIt2261422 CRF07_BC.GZ.2012M325_2012.63

MN426243 BankIt2261422 CRF07_BC.GZ.2012M326_2012.64

MN426244 BankIt2261422 CRF07_BC.GZ.2012M331_2012.65

MN426245 BankIt2261422 CRF07_BC.GZ.2012M333_2012.67

MN426246 BankIt2261422 CRF07_BC.GZ.2012M335_2012.69

MN426247 BankIt2261422 CRF07_BC.GZ.2012M336_2012.69

MN426248 BankIt2261422 CRF07_BC.GZ.2012M339_2012.71

MN426250 BankIt2261422 CRF07_BC.GZ.2012M343_2012.72

MN426251 BankIt2261422 CRF07_BC.GZ.2012M345_2012.73

MN426252 BankIt2261422 CRF07_BC.GZ.2012M346_2012.73

MN425882 BankIt2261420 CRF07_BC.GZ.2008M017_2008.83

MN425883 BankIt2261420 CRF07_BC.GZ.2008M022_2008.45

MN425884 BankIt2261420 CRF07_BC.GZ.2008M026_2008.95

MN425885 BankIt2261420 CRF07_BC.GZ.2008M030_2008.92

MN425886 BankIt2261420 CRF07_BC.GZ.2008M032_2008.93

MN425887 BankIt2261420 CRF07_BC.GZ.2008M036_2008.93

MN425888 BankIt2261420 CRF07_BC.GZ.2008M039_2008.95

MN425890 BankIt2261420 CRF07_BC.GZ.2008M042_2008.99

MN425919 BankIt2261420 CRF07_BC.GZ.2009M002_2009.23

MN425927 BankIt2261420 CRF07_BC.GZ.2009M045_2009.88

MN425961 BankIt2261420 CRF07_BC.GZ.2010M012_2010.96

MN425962 BankIt2261420 CRF07_BC.GZ.2010M013_2010.31

MN425965 BankIt2261420 CRF07_BC.GZ.2010M021_2010.81

MN425967 BankIt2261420 CRF07_BC.GZ.2010M027_2010.46

MN425969 BankIt2261420 CRF07_BC.GZ.2010M032_2010.91

MN425974 BankIt2261420 CRF07_BC.GZ.2010M044_2010.18

MN425975 BankIt2261420 CRF07_BC.GZ.2010M049_2010.83

MN426071 BankIt2261420 CRF07_BC.GZ.2011M125_2011.02

MN426073 BankIt2261420 CRF07_BC.GZ.2011M127_2011.02

MN426080 BankIt2261420 CRF07_BC.GZ.2011M144_2011.3

MN426081 BankIt2261420 CRF07_BC.GZ.2011M145_2011.32

MN426082 BankIt2261420 CRF07_BC.GZ.2011M147_2011.32

MN426086 BankIt2261422 CRF07_BC.GZ.2011M157_2011.39

MN426094 BankIt2261422 CRF07_BC.GZ.2011M166_2011.45

MN426098 BankIt2261422 CRF07_BC.GZ.2011M182_2011.64

MN426100 BankIt2261422 CRF07_BC.GZ.2011M186_2011.67

MN426101 BankIt2261422 CRF07_BC.GZ.2011M187_2011.67

MN426107 BankIt2261422 CRF07_BC.GZ.2011M210_2011.93

MN426108 BankIt2261422 CRF07_BC.GZ.2011M215_2011.93

MN426110 BankIt2261422 CRF07_BC.GZ.2011M217_2011.95

MN426111 BankIt2261422 CRF07_BC.GZ.2011M219_2011.95

MN426112 BankIt2261422 CRF07_BC.GZ.2011M222_2011.97

MN426116 BankIt2261422 CRF07_BC.GZ.2011M229_2012

MN426197 BankIt2261422 CRF07_BC.GZ.2012M209_2012.52

MN426198 BankIt2261422 CRF07_BC.GZ.2012M211_2012.31

MN426199 BankIt2261422 CRF07_BC.GZ.2012M214_2012.32

MN426200 BankIt2261422 CRF07_BC.GZ.2012M218_2012.35

MN426201 BankIt2261422 CRF07_BC.GZ.2012M219_2012.35

MN426202 BankIt2261422 CRF07_BC.GZ.2012M220_2012.33

MN426206 BankIt2261422 CRF07_BC.GZ.2012M229_2012.36

MN426207 BankIt2261422 CRF07_BC.GZ.2012M231_2012.47

MN426210 BankIt2261422 CRF07_BC.GZ.2012M243_2012.41

MN426211 BankIt2261422 CRF07_BC.GZ.2012M247_2012.42

MN426213 BankIt2261422 CRF07_BC.GZ.2012M251_2012.43

MN426217 BankIt2261422 CRF07_BC.GZ.2012M262_2012.45

MN426218 BankIt2261422 CRF07_BC.GZ.2012M264_2012.46

MN426219 BankIt2261422 CRF07_BC.GZ.2012M266_2012.47

MN426220 BankIt2261422 CRF07_BC.GZ.2012M267_2012.47

MN426172 BankIt2261422 CRF07_BC.GZ.2012M134_2012.02

MN426237 BankIt2261422 CRF07_BC.GZ.2012M309_2012.6

MN424787 BankIt2261090 CRF01_AE.GZ.2008M059_2008.67

MN424825 BankIt2261090 CRF01_AE.GZ.2009M093_2009.57

MN424908 BankIt2261090 CRF01_AE.GZ.2011M001_2011.01

MN424942 BankIt2261090 CRF01_AE.GZ.2011M113_2011.87

MN424989 BankIt2261090 CRF01_AE.GZ.2012M011_2012.12

MN424998 BankIt2261090 CRF01_AE.GZ.2012M037_2012.34

MN424999 BankIt2261090 CRF01_AE.GZ.2012M038_2012.34

MN425044 BankIt2261090 CRF01_AE.GZ.2012M172_2012.18

MN424856 BankIt2261090 CRF01_AE.GZ.2010M047_2010.71

MN424862 BankIt2261090 CRF01_AE.GZ.2010M070_2010.22

MN424951 BankIt2261090 CRF01_AE.GZ.2011M148_2011.32

MN424959 BankIt2261090 CRF01_AE.GZ.2011M177_2011.6

MN424968 BankIt2261090 CRF01_AE.GZ.2011M201_2011.85

MN424969 BankIt2261090 CRF01_AE.GZ.2011M203_2011.85

MN424972 BankIt2261090 CRF01_AE.GZ.2011M207_2011.87

MN425058 BankIt2261090 CRF01_AE.GZ.2012M221_2012.35

MN424926 BankIt2261090 CRF01_AE.GZ.2011M057_2011.51

MN424927 BankIt2261090 CRF01_AE.GZ.2011M058_2011.55

MN425007 BankIt2261090 CRF01_AE.GZ.2012M063_2012.55

MN425008 BankIt2261090 CRF01_AE.GZ.2012M067_2012.62

MN424954 BankIt2261090 CRF01_AE.GZ.2011M154_2011.55

MN424895 BankIt2261090 CRF01_AE.GZ.2010M156_2010.77

MN425034 BankIt2261090 CRF01_AE.GZ.2012M145_2012.13

MN425057 BankIt2261090 CRF01_AE.GZ.2012M217_2012.32

MN424818 BankIt2261090 CRF01_AE.GZ.2009M065_2009.33

MN424879 BankIt2261090 CRF01_AE.GZ.2010M116_2010.42

MN425076 BankIt2261090 CRF01_AE.GZ.2012M286_2012.54

MN425093 BankIt2261417 CRF01_AE.GZ.2012M337_2012.69

MN424899 BankIt2261090 CRF01_AE.GZ.2010M174_2010.97

MN424995 BankIt2261090 CRF01_AE.GZ.2012M031_2012.28

MN425041 BankIt2261090 CRF01_AE.GZ.2012M169_2012.17

MN425065 BankIt2261090 CRF01_AE.GZ.2012M239_2012.4

MN424586 BankIt2261090 B.GZ.2008M014_2008.63

MN424917 BankIt2261090 CRF01_AE.GZ.2011M032_2011.36

MN425015 BankIt2261090 CRF01_AE.GZ.2012M091_2012.8

MN424953 BankIt2261090 CRF01_AE.GZ.2011M152_2011.34

MN425049 BankIt2261090 CRF01_AE.GZ.2012M193_2012.26

MN425052 BankIt2261090 CRF01_AE.GZ.2012M207_2012.3

MN424602 BankIt2261090 B.GZ.2009M055_2009.17

MN424587 BankIt2261090 B.GZ.2008M024_2009

MN424913 BankIt2261090 CRF01_AE.GZ.2011M014_2011.15

MN424788 BankIt2261090 CRF01_AE.GZ.2008M067_2008.94

MN425035 BankIt2261090 CRF01_AE.GZ.2012M147_2012.13

MN424773 BankIt2261090 CRF01_AE.GZ.2008M003_2008.63

MN424863 BankIt2261090 CRF01_AE.GZ.2010M074_2010.31

MN425086 BankIt2261417 CRF01_AE.GZ.2012M314_2012.6

MN424632 BankIt2261090 B.GZ.2011M117_2011.91

MN424588 BankIt2261090 B.GZ.2008M028_2008.9

MN424613 BankIt2261090 B.GZ.2010M028_2010.49

MN424601 BankIt2261090 B.GZ.2009M051_2009.97

MN424648 BankIt2261090 B.GZ.2012M316_2012.6

MN425011 BankIt2261090 CRF01_AE.GZ.2012M070_2012.64

MN424829 BankIt2261090 CRF01_AE.GZ.2009M108_2009.69

MN424894 BankIt2261090 CRF01_AE.GZ.2010M150_2010.73

MN424918 BankIt2261090 CRF01_AE.GZ.2011M035_2011.4

MN425013 BankIt2261090 CRF01_AE.GZ.2012M079_2012.72

MN425070 BankIt2261090 CRF01_AE.GZ.2012M256_2012.45

MN424808 BankIt2261090 CRF01_AE.GZ.2009M032_2009.54

MN424991 BankIt2261090 CRF01_AE.GZ.2012M014_2012.15

MN425012 BankIt2261090 CRF01_AE.GZ.2012M076_2012.72

MN425028 BankIt2261090 CRF01_AE.GZ.2012M128_2012.97

MN426249 BankIt2261422 CRF07_BC.GZ.2012M342_2012.72

MN424833 BankIt2261090 CRF01_AE.GZ.2009M122_2009.81

MN424822 BankIt2261090 CRF01_AE.GZ.2009M076_2009.44

MN424835 BankIt2261090 CRF01_AE.GZ.2009M137_2009.93

MN424837 BankIt2261090 CRF01_AE.GZ.2009M147_2010

MN424871 BankIt2261090 CRF01_AE.GZ.2010M101_2010.26

MN424889 BankIt2261090 CRF01_AE.GZ.2010M139_2010.61

MN424997 BankIt2261090 CRF01_AE.GZ.2012M036_2012.31

MN425033 BankIt2261090 CRF01_AE.GZ.2012M144_2012.15

MN424810 BankIt2261090 CRF01_AE.GZ.2009M038_2009.6

MN424975 BankIt2261090 CRF01_AE.GZ.2011M221_2011.99

MN424919 BankIt2261090 CRF01_AE.GZ.2011M038_2011.41

MN424854 BankIt2261090 CRF01_AE.GZ.2010M042_2010.05

MN424890 BankIt2261090 CRF01_AE.GZ.2010M141_2010.64

MN424893 BankIt2261090 CRF01_AE.GZ.2010M149_2010.73

MN424931 BankIt2261090 CRF01_AE.GZ.2011M074_2011.66

MN424934 BankIt2261090 CRF01_AE.GZ.2011M085_2011.74

MN425040 BankIt2261090 CRF01_AE.GZ.2012M161_2012.15

MN425047 BankIt2261090 CRF01_AE.GZ.2012M188_2012.23

MN425069 BankIt2261090 CRF01_AE.GZ.2012M250_2012.43

MN425089 BankIt2261417 CRF01_AE.GZ.2012M319_2012.62

MN425019 BankIt2261090 CRF01_AE.GZ.2012M099_2012.83

MN425043 BankIt2261090 CRF01_AE.GZ.2012M171_2012.18

MN425054 BankIt2261090 CRF01_AE.GZ.2012M213_2012.32

MN425095 BankIt2261417 CRF01_AE.GZ.2012M341_2012.72

MN425010 BankIt2261090 CRF01_AE.GZ.2012M069_2012.64

MN425020 BankIt2261090 CRF01_AE.GZ.2012M100_2012.83

MN424857 BankIt2261090 CRF01_AE.GZ.2010M051_2010.92

MN425061 BankIt2261090 CRF01_AE.GZ.2012M228_2012.35

MN424627 BankIt2261090 B.GZ.2011M053_2011.51

MN424630 BankIt2261090 B.GZ.2011M103_2011.84

MN424631 BankIt2261090 B.GZ.2011M104_2011.84

MN424596 BankIt2261090 B.GZ.2009M025_2009.46

MN424644 BankIt2261090 B.GZ.2012M238_2012.4

MN424961 BankIt2261090 CRF01_AE.GZ.2011M180_2011.62

MN424936 BankIt2261090 CRF01_AE.GZ.2011M092_2011.79

MN424898 BankIt2261090 CRF01_AE.GZ.2010M167_2010.91

MN426221 BankIt2261422 CRF07_BC.GZ.2012M271_2012.52

MN424603 BankIt2261090 B.GZ.2009M070_2009.36

MN424646 BankIt2261090 B.GZ.2012M269_2012.48

MN424604 BankIt2261090 B.GZ.2009M073_2009.4

MN424607 BankIt2261090 B.GZ.2009M095_2009.61

MN424621 BankIt2261090 B.GZ.2010M168_2010.92

MN425930 BankIt2261420 CRF07_BC.GZ.2009M072_2009.4

MN426234 BankIt2261422 CRF07_BC.GZ.2012M304_2012.59

MN426174 BankIt2261422 CRF07_BC.GZ.2012M138_2012.12

MN426079 BankIt2261420 CRF07_BC.GZ.2011M143_2011.31

MN424911 BankIt2261090 CRF01_AE.GZ.2011M008_2011.04

MN424935 BankIt2261090 CRF01_AE.GZ.2011M086_2011.74

MN424859 BankIt2261090 CRF01_AE.GZ.2010M059_2010.43

MN424964 BankIt2261090 CRF01_AE.GZ.2011M190_2011.71

MN424882 BankIt2261090 CRF01_AE.GZ.2010M122_2010.46

MN424610 BankIt2261090 B.GZ.2009M135_2009.92

MN424996 BankIt2261090 CRF01_AE.GZ.2012M035_2012.3

MN424617 BankIt2261090 B.GZ.2010M110_2010.36

MN424622 BankIt2261090 B.GZ.2011M006_2011.03

MN424623 BankIt2261090 B.GZ.2011M020_2011.23

MN424614 BankIt2261090 B.GZ.2010M048_2010.82

MN424635 BankIt2261090 B.GZ.2011M213_2011.93

MN424615 BankIt2261090 B.GZ.2010M052_2010.96

MN424877 BankIt2261090 CRF01_AE.GZ.2010M113_2010.4

MN425045 BankIt2261090 CRF01_AE.GZ.2012M181_2012.2

MN424881 BankIt2261090 CRF01_AE.GZ.2010M118_2010.44

MN424618 BankIt2261090 B.GZ.2010M121_2010.44

MN424649 BankIt2261090 B.GZ.2012M329_2012.64

MN424896 BankIt2261090 CRF01_AE.GZ.2010M157_2010.78

MN425067 BankIt2261090 CRF01_AE.GZ.2012M242_2012.43

MN425075 BankIt2261090 CRF01_AE.GZ.2012M285_2012.54

MN425005 BankIt2261090 CRF01_AE.GZ.2012M052_2012.43

MN424820 BankIt2261090 CRF01_AE.GZ.2009M074_2009.44

MN424872 BankIt2261090 CRF01_AE.GZ.2010M102_2010.28

MN424876 BankIt2261090 CRF01_AE.GZ.2010M112_2010.38

MN424878 BankIt2261090 CRF01_AE.GZ.2010M114_2010.42

MN424884 BankIt2261090 CRF01_AE.GZ.2010M130_2010.55

MN424930 BankIt2261090 CRF01_AE.GZ.2011M073_2011.66

MN425006 BankIt2261090 CRF01_AE.GZ.2012M057_2012.51

MN424809 BankIt2261090 CRF01_AE.GZ.2009M034_2009.27

MN424955 BankIt2261090 CRF01_AE.GZ.2011M163_2011.41

MN424966 BankIt2261090 CRF01_AE.GZ.2011M197_2011.83

MN424949 BankIt2261090 CRF01_AE.GZ.2011M136_2011.25

MN424958 BankIt2261090 CRF01_AE.GZ.2011M173_2011.58

MN425066 BankIt2261090 CRF01_AE.GZ.2012M241_2012.41

MN425099 BankIt2261417 CRF01_AE.GZ.2012M349_2012.01

MN424880 BankIt2261090 CRF01_AE.GZ.2010M117_2010.42

MN425018 BankIt2261090 CRF01_AE.GZ.2012M097_2012.83

MN425060 BankIt2261090 CRF01_AE.GZ.2012M227_2012.35

MN424633 BankIt2261090 B.GZ.2011M171_2011.59

MN424647 BankIt2261090 B.GZ.2012M280_2012.52

MN424634 BankIt2261090 B.GZ.2011M208_2011.89

MN425064 BankIt2261090 CRF01_AE.GZ.2012M235_2012.38

MN427005 BankIt2261423 CRF55_01B.GZ.2008M012_2008.31

MN427006 BankIt2261423 CRF55_01B.GZ.2008M015_2008.64

MN427009 BankIt2261423 CRF55_01B.GZ.2008M043_2008.96

MN427010 BankIt2261423 CRF55_01B.GZ.2008M049_2008.24

MN427011 BankIt2261423 CRF55_01B.GZ.2008M063_2008.79

MN427012 BankIt2261423 CRF55_01B.GZ.2008M066_2008.94

MN427021 BankIt2261423 CRF55_01B.GZ.2009M054_2009.13

MN427022 BankIt2261423 CRF55_01B.GZ.2009M063_2009.32

MN427023 BankIt2261423 CRF55_01B.GZ.2009M081_2009.5

MN427025 BankIt2261423 CRF55_01B.GZ.2009M097_2009.61

MN427027 BankIt2261423 CRF55_01B.GZ.2009M102_2009.65

MN427028 BankIt2261423 CRF55_01B.GZ.2009M103_2009.65

MN427029 BankIt2261423 CRF55_01B.GZ.2009M106_2009.67

MN427030 BankIt2261423 CRF55_01B.GZ.2009M107_2009.67

MN427031 BankIt2261423 CRF55_01B.GZ.2009M109_2009.69

MN427032 BankIt2261423 CRF55_01B.GZ.2009M117_2009.74

MN427033 BankIt2261423 CRF55_01B.GZ.2009M121_2009.81

MN427034 BankIt2261423 CRF55_01B.GZ.2009M127_2009.86

MN427037 BankIt2261423 CRF55_01B.GZ.2009M141_2009.93

MN427038 BankIt2261423 CRF55_01B.GZ.2009M144_2009.97

MN427058 BankIt2261423 CRF55_01B.GZ.2010M087_2010.03

MN427059 BankIt2261423 CRF55_01B.GZ.2010M100_2010.24

MN427061 BankIt2261423 CRF55_01B.GZ.2010M126_2010.51

MN427062 BankIt2261423 CRF55_01B.GZ.2010M129_2010.53

MN427063 BankIt2261423 CRF55_01B.GZ.2010M144_2010.68

MN427064 BankIt2261423 CRF55_01B.GZ.2010M145_2010.69

MN427065 BankIt2261423 CRF55_01B.GZ.2010M146_2010.69

MN427066 BankIt2261423 CRF55_01B.GZ.2010M153_2010.73

MN427067 BankIt2261423 CRF55_01B.GZ.2010M154_2010.73

MN427068 BankIt2261423 CRF55_01B.GZ.2010M159_2010.79

MN427069 BankIt2261423 CRF55_01B.GZ.2010M164_2010.88

MN427070 BankIt2261423 CRF55_01B.GZ.2010M166_2010.9

MN427071 BankIt2261423 CRF55_01B.GZ.2010M169_2010.93

MN427072 BankIt2261423 CRF55_01B.GZ.2010M171_2010.95

MN427073 BankIt2261423 CRF55_01B.GZ.2011M021_2011.23

MN427074 BankIt2261423 CRF55_01B.GZ.2011M028_2011.32

MN427075 BankIt2261423 CRF55_01B.GZ.2011M039_2011.43

MN427076 BankIt2261423 CRF55_01B.GZ.2011M051_2011.5

MN427077 BankIt2261423 CRF55_01B.GZ.2011M052_2011.51

MN427078 BankIt2261423 CRF55_01B.GZ.2011M066_2011.61

MN427079 BankIt2261423 CRF55_01B.GZ.2011M071_2011.64

MN427080 BankIt2261423 CRF55_01B.GZ.2011M081_2011.7

MN427081 BankIt2261423 CRF55_01B.GZ.2011M115_2011.91

MN427091 BankIt2261425 CRF55_01B.GZ.2012M004_2012.04

MN427092 BankIt2261425 CRF55_01B.GZ.2012M012_2012.14

MN427093 BankIt2261425 CRF55_01B.GZ.2012M039_2012.34

MN427094 BankIt2261425 CRF55_01B.GZ.2012M045_2012.38

MN427095 BankIt2261425 CRF55_01B.GZ.2012M061_2012.54

MN427096 BankIt2261425 CRF55_01B.GZ.2012M065_2012.55

MN427097 BankIt2261425 CRF55_01B.GZ.2012M074_2012.66

MN427098 BankIt2261425 CRF55_01B.GZ.2012M077_2012.71

MN427099 BankIt2261425 CRF55_01B.GZ.2012M080_2012.72

MN427100 BankIt2261425 CRF55_01B.GZ.2012M081_2012.72

MN427101 BankIt2261425 CRF55_01B.GZ.2012M084_2012.74

MN427102 BankIt2261425 CRF55_01B.GZ.2012M087_2012.78

MN427104 BankIt2261425 CRF55_01B.GZ.2012M095_2012.81

MN427105 BankIt2261425 CRF55_01B.GZ.2012M105_2012.87

MN427106 BankIt2261425 CRF55_01B.GZ.2012M120_2012.93

MN427107 BankIt2261425 CRF55_01B.GZ.2012M127_2012.97

MN427108 BankIt2261425 CRF55_01B.GZ.2012M130_2012.01

MN427109 BankIt2261425 CRF55_01B.GZ.2012M177_2012.19

MN427110 BankIt2261425 CRF55_01B.GZ.2012M178_2012.19

MN427111 BankIt2261425 CRF55_01B.GZ.2012M182_2012.2

MN427113 BankIt2261425 CRF55_01B.GZ.2012M201_2012.29

MN427114 BankIt2261425 CRF55_01B.GZ.2012M204_2012.29

MN427007 BankIt2261423 CRF55_01B.GZ.2008M023_2008.46

MN427014 BankIt2261423 CRF55_01B.GZ.2009M008_2009.48

MN427015 BankIt2261423 CRF55_01B.GZ.2009M012_2009.15

MN427017 BankIt2261423 CRF55_01B.GZ.2009M014_2009.62

MN427019 BankIt2261423 CRF55_01B.GZ.2009M023_2009.46

MN427020 BankIt2261423 CRF55_01B.GZ.2009M026_2009.47

MN427041 BankIt2261423 CRF55_01B.GZ.2010M005_2010.45

MN427048 BankIt2261423 CRF55_01B.GZ.2010M039_2010.92

MN427050 BankIt2261423 CRF55_01B.GZ.2010M054_2010.2

MN427054 BankIt2261423 CRF55_01B.GZ.2010M071_2010.24

MN427055 BankIt2261423 CRF55_01B.GZ.2010M073_2010.29

MN427056 BankIt2261423 CRF55_01B.GZ.2010M075_2010.35

MN427082 BankIt2261423 CRF55_01B.GZ.2011M130_2011.16

MN427083 BankIt2261425 CRF55_01B.GZ.2011M141_2011.31

MN427084 BankIt2261425 CRF55_01B.GZ.2011M142_2011.31

MN427085 BankIt2261425 CRF55_01B.GZ.2011M153_2011.34

MN427087 BankIt2261425 CRF55_01B.GZ.2011M181_2011.62

MN427089 BankIt2261425 CRF55_01B.GZ.2011M220_2011.95

MN427116 BankIt2261425 CRF55_01B.GZ.2012M224_2012.35

MN427117 BankIt2261425 CRF55_01B.GZ.2012M233_2012.38

MN427118 BankIt2261425 CRF55_01B.GZ.2012M237_2012.4

MN427119 BankIt2261425 CRF55_01B.GZ.2012M244_2012.41

MN427123 BankIt2261425 CRF55_01B.GZ.2012M259_2012.45

MN427124 BankIt2261425 CRF55_01B.GZ.2012M270_2012.52

MN427125 BankIt2261425 CRF55_01B.GZ.2012M273_2012.51

MN427126 BankIt2261425 CRF55_01B.GZ.2012M274_2012.53

MN427127 BankIt2261425 CRF55_01B.GZ.2012M275_2012.51

MN427128 BankIt2261425 CRF55_01B.GZ.2012M295_2012.56

MN427129 BankIt2261425 CRF55_01B.GZ.2012M297_2012.57

MN427131 BankIt2261425 CRF55_01B.GZ.2012M307_2012.59

MN427135 BankIt2261425 CRF55_01B.GZ.2012M330_2012.65

MN427138 BankIt2261425 CRF55_01B.GZ.2012M350_2012.02

MN427008 BankIt2261423 CRF55_01B.GZ.2008M034_2008.93

MN427016 BankIt2261423 CRF55_01B.GZ.2009M013_2009.18

MN427045 BankIt2261423 CRF55_01B.GZ.2010M014_2010.31

MN427049 BankIt2261423 CRF55_01B.GZ.2010M053_2010.96

MN427051 BankIt2261423 CRF55_01B.GZ.2010M062_2010.58

MN427086 BankIt2261425 CRF55_01B.GZ.2011M175_2011.59

MN427088 BankIt2261425 CRF55_01B.GZ.2011M191_2011.73

MN427121 BankIt2261425 CRF55_01B.GZ.2012M253_2012.44

MN427122 BankIt2261425 CRF55_01B.GZ.2012M255_2012.45

MN427130 BankIt2261425 CRF55_01B.GZ.2012M302_2012.6

MN427133 BankIt2261425 CRF55_01B.GZ.2012M320_2012.62

MN427136 BankIt2261425 CRF55_01B.GZ.2012M332_2012.65

MN427137 BankIt2261425 CRF55_01B.GZ.2012M340_2012.72

MN424817 BankIt2261090 CRF01_AE.GZ.2009M058_2009.19

MN424887 BankIt2261090 CRF01_AE.GZ.2010M136_2010.59

MN424994 BankIt2261090 CRF01_AE.GZ.2012M030_2012.28

MN425068 BankIt2261090 CRF01_AE.GZ.2012M249_2012.43

MN425999 BankIt2261420 CRF07_BC.GZ.2010M158_2010.79

MN427115 BankIt2261425 CRF55_01B.GZ.2012M210_2012.35

MN427112 BankIt2261425 CRF55_01B.GZ.2012M196_2012.27

MN425084 BankIt2261417 CRF01_AE.GZ.2012M303_2012.58

MN425100 BankIt2261417 CRF01_AE.GZ.2012M351_2012.5

MN424912 BankIt2261090 CRF01_AE.GZ.2011M011_2011.05

MN425025 BankIt2261090 CRF01_AE.GZ.2012M113_2012.91

MN424922 BankIt2261090 CRF01_AE.GZ.2011M050_2011.49

MN424928 BankIt2261090 CRF01_AE.GZ.2011M064_2011.57

MN424986 BankIt2261090 CRF01_AE.GZ.2012M007_2012.1

MN425097 BankIt2261417 CRF01_AE.GZ.2012M347_2012.73

MN424939 BankIt2261090 CRF01_AE.GZ.2011M106_2011.85

MN424941 BankIt2261090 CRF01_AE.GZ.2011M112_2011.87

MN424943 BankIt2261090 CRF01_AE.GZ.2011M114_2011.88

MN424774 BankIt2261090 CRF01_AE.GZ.2008M005_2008.59

MN424775 BankIt2261090 CRF01_AE.GZ.2008M007_2008.61

MN424776 BankIt2261090 CRF01_AE.GZ.2008M008_2008.61

MN425081 BankIt2261090 CRF01_AE.GZ.2012M299_2012.58

MN424957 BankIt2261090 CRF01_AE.GZ.2011M169_2011.54

MN425009 BankIt2261090 CRF01_AE.GZ.2012M068_2012.64

MN424777 BankIt2261090 CRF01_AE.GZ.2008M020_2008.36

MN424804 BankIt2261090 CRF01_AE.GZ.2009M001_2009.22

MN424962 BankIt2261090 CRF01_AE.GZ.2011M184_2011.66
